# Darker ants dominate the canopy: Testing macroecological hypotheses for patterns in colour along a microclimatic gradient

**DOI:** 10.1111/1365-2656.13110

**Published:** 2019-10-21

**Authors:** Stephanie J. Law, Tom R. Bishop, Paul Eggleton, Hannah Griffiths, Louise Ashton, Catherine Parr

**Affiliations:** ^1^ Department of Earth, Ocean and Ecological Sciences School of Environmental Sciences University of Liverpool Liverpool UK; ^2^ Department of Zoology and Entomology University of Pretoria Pretoria South Africa; ^3^ Life Sciences Department Natural History Museum London UK; ^4^ School of Biological Sciences The University of Hong Kong Hong Kong China; ^5^ School of Animal, Plant and Environmental Sciences University of the Witwatersrand Johannesburg South Africa

**Keywords:** colouration, Gloger's rule, macroecology, melanism‐desiccation, thermal melanism, tropical forest, ultraviolet‐B radiation, vertical stratification

## Abstract

1. Gradients in cuticle lightness of ectotherms have been demonstrated across latitudes and elevations. Three key hypotheses have been used to explain these macroecological patterns: the thermal melanism hypothesis (TMH), the melanism‐desiccation hypothesis (MDH) and the photo‐protection hypothesis (PPH). Yet the broad abiotic measures, such as temperature, humidity and UV‐B radiation, typically used to detect these ecogeographical patterns, are a poor indication of the microenvironment experienced by small, cursorial ectotherms like ants.
2. We tested whether these macroecological hypotheses explaining cuticle lightness held at habitat and microclimatic level by using a vertical gradient within a tropical rainforest.
3. We sampled 222 ant species in lowland, tropical rainforest across four vertical strata: subterranean, ground, understory and canopy. We recorded cuticle lightness, abundance and estimated body size for each species and calculated an assemblage‐weighted mean for cuticle lightness and body size for each vertical stratum. Abiotic variables (air temperature, vapour pressure deficit and UV‐B radiation) were recorded for each vertical stratum.
4. We found that cuticle lightness of ant assemblages was vertically stratified: ant assemblages in the canopy and understory were twice as dark as assemblages in ground and subterranean strata. Cuticle lightness was not correlated with body size, and there was no support for the TMH. Rather, we attribute this cline in cuticle lightness to a combination of the MDH and the PPH.
5. Our findings indicate that broad macroecological patterns can be detected at much smaller spatial scales and that microclimatic gradients can shape trait variation, specifically the cuticle lightness of ants. These results suggest that any changes to microclimate that occur due to land‐use change or climate warming could drive selection of ants based on cuticle colour, altering assemblage structure and potentially ecosystem functioning.

## Graphical abstract

This article is the first to show evidence for the vertical stratification of melanism in tropical ant assemblages. The authors take a novel approach and test traditional macroecology findings at a much smaller scale. Using macroecological ideas but with microclimatic data, they advance the generality of our understanding of ectotherm colouration.

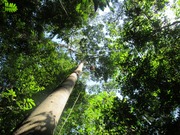

## 1 INTRODUCTION

Colour variation in organisms is present at a range of geographical and ecological scales from the dark and light polymorphisms of the peppered moth (Grant, Cook, Clarke, & Owen, 1998) to the striking and bright colour variations of the strawberry poison frog (Siddiqi, Cronin, Loew, Vorobyev, & Summers, 2004). Such variation is driven by diverse abiotic and biotic pressures including protection from predators through camouflage and mimicry, sexual selection, pathogen resistance and climatic gradients (Clusella Trullas, van Wyk, & Spotila, 2007; True, 2003).

A substantial body of macroecological work exists linking colour variation in insects to a range of hypotheses at both the intraspecific and interspecific level (Clusella Trullas et al., 2007; True, 2003). Yet studies on colour variation at a smaller, microecological, scale are scarce. With this in mind, here we investigate colour variation in ant assemblages within different tropical forest microhabitats.

Three key hypotheses are used to explain variation in colour, and all principally refer to patterns of achromatic variation (light to dark). While integument colour is determined by both structural features and the deposition of a variety of pigments (Shawkey & D'Alba, 2017), melanin is the most common pigment in animals and is largely responsible for patterns of achromatic variation (Fuzeau‐Braesch, 1972; Shawkey & D'Alba, 2017; True, 2003).

First, the thermal melanism hypothesis (TMH) predicts that cooler environments, often at higher altitudes and latitudes, favour darker individuals with more melanin (Bogert, 1949; Clusella Trullas et al., 2007). Darker individuals are able to absorb solar radiation more quickly through lower reflectance and reach higher temperatures than lighter individuals of the same size exposed to the same conditions (Clusella Trullas et al., 2007; Willmer & Unwin, 1981). Melanism ultimately leads to greater fitness levels in cool environments as reaching operating temperatures more quickly benefits the individual through longer activity times and greater performance (Clusella Trullas et al., 2007; Huey & Kingsolver, 1989). Clines in melanism have been found in dragonflies, wasps and ants, whereby darker individuals are more often found or are more abundant in cooler environments (Bishop et al., 2016; De Souza, Turillazzi, Lino‐Neto, & Santini, 2017; Zeuss, Brandl, Brändle, Rahbek, & Brunzel, 2014).

Second, the melanism‐desiccation hypothesis (MDH) predicts that darker individuals will be more prevalent in drier environments. Increased melanization can provide greater resistance to desiccation by decreasing cuticular permeability, which in turn reduces cuticular water loss (Kalmus, 1941). Melanin as a barrier to water loss has not been considered for many taxa but it has been widely tested in populations of *Drosophila* (Parkash, Ramniwas, Rajpurohit, & Sharma, 2008; Parkash, Singh, & Ramniwas, 2009). Darker individuals of *Drosophila* and *Hemideina* (tree weta; Orthoptera: Anostostomatidae) have performed better than lighter ones under desiccation stress in experimental laboratory conditions (King & Sinclair, 2015; Parkash et al., 2008, 2009).

Third, Gloger's rule predicts an increase in melanization (and hence darker colours) in warmer and more humid environments (Rensch, 1929), the opposite of the pattern predicted by the TMH and the MDH. While the mechanistic underpinnings of the TMH and the MDH are well defined (Clusella Trullas et al., 2007; Kalmus, 1941), the proposed mechanisms driving Gloger's rule are varied but, include camouflage, protection against parasites and pathogens, and protection against UV‐B radiation (or photo‐protection) (Delhey, 2019). Increased melanization correlates with greater UV‐B radiation for *Drosophila*, ants and plants (Bastide, Yassin, Johanning, & Pool, 2014; Bishop et al., 2016; Koski & Ashman, 2015). Furthermore, it has been shown experimentally that ultraviolet radiation has deleterious effects on the fitness of ectotherms (Wang, Liu, Wang, Du, & Deng, 2008) and that higher levels of melanin are able to protect against this harmful ultraviolet radiation (Mosse & Lyakh, 1994). As such, in this study we focus on the protection melanin provides against UV‐B radiation as a potential driving mechanism behind patterns in melanization and refer to this as the photo‐protection hypothesis (PPH).

The ability of an individual to regulate body temperature or reduce water loss is not solely dependent on colour. Body size is a critical factor in determining heat loss and water loss. In cool environments, larger individuals benefit by losing heat less rapidly than smaller bodies (Porter & Gates, 1969; Stevenson, 1985) but it also means that heat gain is slower. While in dry environments, larger individuals lose less water due to their lower surface area to volume ratios (Hadley, 1994). It is therefore expected that body size and colour interact in thermoregulation and desiccation resistance. Thus, investigations of the TMH and the MDH should consider differences in body size. For example, the greater thermal inertia of large organisms can be offset if the individual is also dark as solar radiation is absorbed more quickly. This interaction has been documented in field studies of ectotherms (focusing on thermoregulation) with most finding a positive relationship between body size and degree of melanization (Bishop et al., 2016; Schweiger & Beierkuhnlein, 2016).

These ecogeographical hypotheses used to describe clines in biological traits, such as colour and body size, have typically been tested over large geographical scales using macroclimatic data (Bishop et al., 2016; Zeuss et al., 2014). However, most organisms do not experience the same macroclimatic conditions used in the simple description of species distributions; rather, they live in highly heterogeneous microclimates (Potter, Woods, & Pincebourde, 2013). For small ectotherms, such as insects, microclimatic gradients are especially important for thermoregulation (Kaspari, Clay, Lucas, Yanoviak, & Kay, 2015; Pincebourde & Casas, 2015; Stark, Adams, Fredley, & Yanoviak, 2017). For example, small organisms like ants that live close to the surface, can experience temperatures up to 5°C higher than organisms just a few millimetres taller, as exposed surfaces can become superheated relative to the air 1–3 mm above them (Kaspari et al., 2015; Stark et al., 2017).

In tropical forests, the climatic variables that influence mechanisms of heat gain and loss, such as solar radiation, air temperature and humidity (Porter & Gates, 1969; Stevenson, 1985), all differ within a short geographical space both within and between microhabitats (Kaspari et al., 2015; Mark & Ashton, 1992). In particular, a vertical gradient in these climatic variables exists from the forest floor to the canopy (Madigosky, 2004). The canopy is often subject to higher temperatures, lower humidity and higher levels of UV‐B radiation. This climatic gradient along vertical strata can be steeper than the climatic gradients observed across elevation and latitude; in tropical rainforest, on average, temperature drops by 0.7°C with every 100 m increase in elevation but can vary by 2.2°C over 20 m between the ground and canopy (Scheffers et al., 2013). This large vertical climatic gradient makes it a good candidate to test these ecogeographical rules at a much smaller spatial scale.

**Figure 1:**
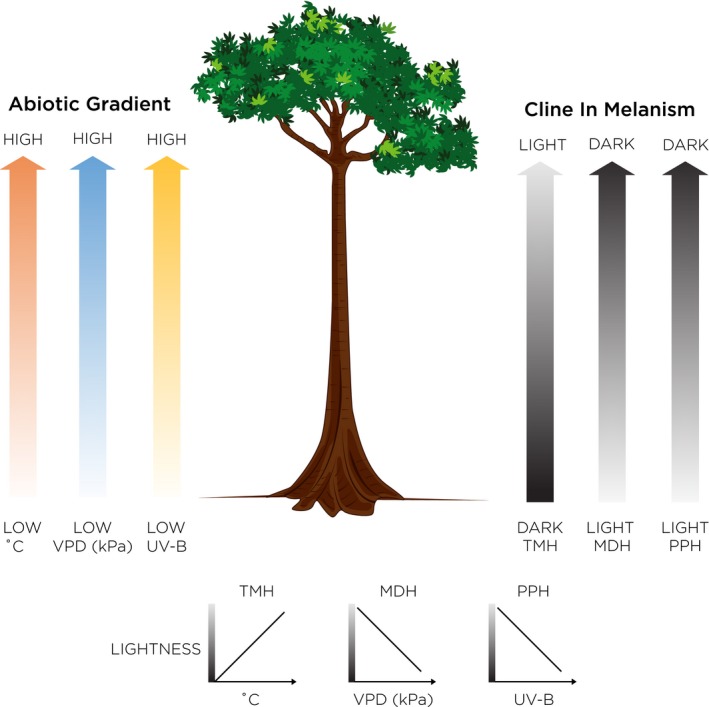
Image shows predictions for vertical gradients in abiotic factors (temperature, vapour pressure deficit (VPD) and UV‐B radiation) and in melanism according to the three hypotheses (MDH, melanism‐desiccation; PPH, photo‐protection; TMH, thermal melanism). VPD is a measure of the drying power of the air; desiccation is greater in environments with a high VPD

According to the TMH, we would expect cuticle colour to get lighter as temperature increases towards the canopy. Conversely, if the MDH or the PPH is driving patterns in colour, we would expect cuticle colour to get darker as vapour pressure deficit (VPD, the drying power of the air) increases and UV‐B radiation increases towards the canopy (Figure 1). Differences in these predictions allow us to disentangle the influence of the TMH over the MDH and the PPH; however, the potential collinearity of VPD and UV‐B in this study system does not allow the MDH to be easily distinguished from the PPH. Understanding the mechanism that may be determining colour patterns has become increasingly important as abiotic factors such as temperature, VPD and UV‐B are predicted to change due to climate change but also with deforestation and the conversion of tropical forest to agricultural land (Brown, 1993; Roulin, 2014; Senior, Hill, González del Pliego, Goode, & Edwards, 2017).

In order to determine which colour is successful under a given set of environmental conditions, it is useful to account for changes in the relative abundance of species and examine assemblage level variation. While the spatial scale may be small it is possible to take a macroecological approach by focusing on aggregations of species in certain locations (McGill, 2019). Furthermore, by calculating an assemblage‐weighted mean the idiosyncrasies of species can be avoided and general patterns and principles detected (Millien et al., 2006). The significance of trait variation in assemblages and the extent that microclimate may influence established ecogeographical patterns have both been identified as major challenges in the field of macroecology (Chown & Gaston, 2016). We address this knowledge gap by (a) describing spatial patterns of cuticle colour in ant (Hymenoptera: Formicidae) assemblages across a vertical microclimatic gradient; and (b) testing to what extent these patterns of colour, and body size, fit predictions of the TMH, the MDH and the PPH.

## 2 MATERIALS AND METHODS

### 2.1 Study site and ant collection

Ant assemblage data were collected in lowland, old‐growth dipterocarp rainforest in Maliau Basin Conservation Area, Sabah, Malaysia (4°44′35″ to 55″N and 116°58′10″–30″) (Law et al., 2019). Elevation of the study site was 270 m a.s.l and, over 2013–2018, mean monthly temperature was 24.9°C ± 3.1°C, mean monthly rainfall 165 mm ± 70 mm and mean monthly humidity 77% ± 16% (Belian Meteorological Station, Maliau Basin Conservation Area). Ants were sampled across four vertical strata (subterranean, ground, understory and canopy) in four plots; each plot was 50 m × 50 m and spaced at least 100 m apart.

Subterranean and arboreal ants were collected from February to May 2016 while ground ants were collected during June and July 2014. A variety of trapping techniques were used. Subterranean ants were collected at a depth of 24 cm using traps baited with a mixture of honey and tuna, ground ants using Winkler bag extractions and arboreal ants using baited traps similar to the baited pitfall trap method described by Yusah, Fayle, Harris, and Foster (2012). Arboreal traps were baited with carbohydrate and protein, placed at 5 m vertical intervals on three trees within each plot and left for 24 hr. The stratum (canopy or understory) of each trap location was recorded; traps placed above the first branch were identified as the canopy and traps below the first branch as the understory. Tree selection based on species or absolute height was not possible due to the limited number of trees safe to climb within each 50 m × 50 m plot. However, all trees were either emergent or reached the high canopy and belonged to the genera *Parashorea* or *Shorea*. In total, across four plots data were collected from the following: 120 subterranean traps, 28 Winkler bag extractions, 272 understory traps and 144 canopy traps. See Appendix S1 for details of all ant collection methods.

On collection, specimens from all strata were transferred to 70% ethanol. All ant specimens were identified to genus (Fayle, Yusah, & Hashimoto, 2014) and to morphospecies level. Voucher specimens are lodged in a reference collection at the University of Liverpool.

### 2.2 Lightness data

The colour of each ant species was classified categorically by eye using a set pre‐determined colours (see Appendix S2; see Bishop et al., 2016 and Parr et al., 2017 for method). Colouration of the hairs was ignored, and the assigned colour was focused solely on the cuticle. The colour of the head, mesosoma and gaster was recorded for up to 12 individuals of every ant species found within each stratum (mean = 4.6 specimens for each species). Some species in the dataset were rare, and only one or a few individuals were collected. A single dominant colour was assigned for each species, this was determined as the modal colour across all body parts and individuals for each species. Each colour was linked to a set of RGB (red, green and blue) values which were extracted from the original colour wheel using the image editing software paint.net (v.4.0.3). Using the *‘*rgb2hsv’ function in r, RGB values for each colour were converted into HSV (hue, saturation and value) format. The HSV scale is a common cylindrical‐coordinate representation of colour where hue describes the dominant wavelength, saturation defines the intensity of the hue present and value refers to the lightness of a colour. We analyse only lightness (v, in HSV) of the dominant colour of each species. Lightness of the dominant colour strongly correlates with mean colour for each species (Spearman's *ρ* = 0.97, *p* < 0001; Appendix S2). All analyses are based on worker castes: only minor workers were measured for colour and body size.

Only one person (the lead author, SL) recorded colour for all target ant specimens; however, we tested the method for observational error by asking 16 people to assign colour to a standardized set of 71 photographs from AntWeb (http://antweb.org/). Observational error was low, and there was a strong correlation between the authors estimate for lightness and the mean estimate (Appendix S2) suggesting reasonable precision in colour assignment. Additionally, we tested for intraspecific variation on a subsample of 20 species. Lightness values were estimated for 50 specimens of each species; intraspecific variation was small with the mean coefficient of variation estimated from the 20 species averaging 0.053 for the head, 0.089 for the mesosoma and 0.115 for the gaster (Appendix S2).

### 2.3 Estimating body size

For the same individuals, we estimated body size using a standard measurement called Weber's length (hereafter called body size) (mean = 4.6 specimens for each species). This is the distance between the anterodorsal margin of the pronotum and the posteroventral margin of the propodeum (Brown, 1953). Using ocular micrometres attached to stereomicroscopes, Weber's length was recorded to the nearest 0.01 mm. Ant specimens were measured using the highest level of magnification that allowed the entire mesosoma of the specimen to be fitted under the range of the ocular micrometre. We again tested for intraspecific variation in body size on a subset of 20 species; variation was low with a mean coefficient of variation of 0.053 (Appendix S2).

Physical specimens were not available for nine of the species originally caught in traps; this was due to damage to specimens because some specimens had been returned as reference specimens to the Universiti Malaysia Sabah before colour and body size was recorded, and for some species, only major workers were collected. These species were rare, and a few individuals were sampled; they were omitted from the analysis.

### 2.4 Abiotic data

Estimates of air temperature and relative humidity were recorded for ground, understory and canopy strata during February to May 2016 (Law, Ashton, Griffiths, Eggleton, & Parr, 2018). Climate measurements were recorded by placing data loggers (Thermocron ibuttons^®^, model DS1923) at 5 m vertical intervals from the ground to the canopy on the largest sampled tree within each plot. Climatic values for each stratum were calculated using only the data loggers placed within the range of each stratum. In October 2016, soil temperature was recorded at 25 points within each plot using a digital temperature probe at an approximate depth of 10 cm (Law et al., 2019).

Vapour pressure deficit (VPD) is a measure of the drying power of the air and is more biologically relevant than relative humidity alone as it relies on both temperature and humidity (Anderson, 1936). Using our measurements of temperature and humidity, we calculated VPD applying the following formulae (Monteith & Unsworth, 2007; Murray, 1967):

$$\text{Saturated}\;\text{vapour}\;\text{pressure}\;\text{(SVP)}=610.7\;\times \;10^{\left(7.5\times T/237.3+T\right)}$$

$$\text{Vapour}\;\text{pressure}\;\text{deficit}\;\text{(VPD)}=\left(100-\text{RH}/100\right)\;\times \;\text{SVP}$$

where RH was relative humidity (%), T was temperature (°C) and the values 7.5 and 237.3 are constants. As relative humidity for subterranean strata was not recorded, we assigned a humidity value of 99% to each subterranean stratum. This figure was chosen to be slightly higher than the mean humidity we recorded at ground level and because humidity in moist soils (like the tropics) is almost always near 100% (Bittelli, Campbell, & Tomei, 2015).

UV‐B radiation was measured during October–November 2018 (Law et al., 2019), on the same trees and at the same 5 m vertical intervals as temperature and humidity. Although UV‐B radiation was recorded at a different date to ant collection, seasonal variation in UV‐B radiation at low latitudes is minimal (Beckmann et al., 2014; Appendix S3). All readings were taken within 1 hr of solar noon using a Solarmeter^®^ (Model 6.0) that responds to wavelengths between 235 and 330 nm. Although these wavelengths are within the lower range of UV‐A (315–400 nm) and upper range of UV‐C (100–280 nm), the Solarmeter^®^ is most sensitive to wavelengths within the UV‐B range (280–315 nm) as such we refer to only UV‐B radiation. Additionally, UV‐B was recorded at 12 random points on the ground within each plot to estimate UV‐B at ground level and a further 12 readings were taken in a clear, open area with no obstructions to the sky; the latter was used as a proxy for incident radiation on top of the canopy. Transmittance of UV‐B radiation was defined as the irradiance recorded (at a specific height) divided by the incident radiation (clear sky). It was assumed that transmittance of UV‐B radiation to the subterranean stratum was zero. See Appendix S1 for details of all methods.

### 2.5 Statistical analysis

All data manipulation and analyses were carried out using R version 3.4.4 (R Core Team, 2018). Apart from testing for phylogenetic signal at the genus level (see Appendix S4), all analyses are performed at the assemblage level.

Assemblage‐weighted means (AWM) of lightness and body size were calculated for each assemblage (*n* = 16:4 plots × 4 strata). AWMs were calculated by:

$$\text{AWM}=\sum_{i}^{S}p_{i}x_{i}$$

where *S* is the number of species in an assemblage, *p_i_* is the proportional abundance of each species and *x_i_* is the trait value (lightness or body size) of each species.

Linear mixed models in the ‘lme4’ package (Bates, Mächler, Bolker, & Walker, 2015) were used to assess how much variation in assemblage‐weighted lightness could be explained by stratum and by assemblage‐weighted body size. To account for spatial correlation of assemblages from different strata sampled within the same plot, we included plot as a random factor. The response variable of assemblage‐weighted lightness was logit transformed to meet Gaussian assumptions as recommended for proportional data (Warton & Hui, 2011). Visual inspection of residual plots did not reveal any obvious deviations from homoscedasticity or normality. An information‐theoretic approach was used to arrive at the best descriptive model (Burnham & Anderson, 2002), using bias‐corrected Akaike information criterion (AICc) (Hurvich & Tsai, 1989). To evaluate the variance of the data explained by each model, we calculated marginal (fixed effects) and conditional (fixed and random effects) *R* ^2^, according to Nakagawa and Schielzeth (2013) using the ‘r.squaredGLMM’ function in the package ‘mumin’ (Barton, 2018). To determine which strata differed in assemblage‐weighted lightness, pairwise comparisons were calculated for the best descriptive model based on differences of least square means (function ‘difflsmeans’, package ‘lmertest’, Kuznetsova, Brockhoff, & Christensen, 2017).

Across the plots, the distribution of lightness is not evenly spread (Appendix S5). If the pool of species is biased towards one end of the lightness spectrum, then a difference in species richness could lead to differences in colour. For example, an assemblage may be dark in colour simply because a large proportion of the species available to colonize it are themselves dark. To examine the influence of species richness on the lightness value of the assemblage, a linear mixed model of assemblage‐weighted lightness as a function of assemblage species richness was run, specifying plot as a random factor.

A further analysis was carried out to disentangle which hypothesis (TMH, MDH or PPH) best explains the spatial pattern in assemblage‐weighted lightness. We constructed linear mixed models for each hypothesis using the same response variable (assemblage‐weighted lightness) and random effects structure (tree nested within plot). For the TMH, both temperature and assemblage‐weighted body size, and temperature alone, were used as explanatory variables; for the MDH, both VPD and assemblage‐weighted body size, and VPD alone, were set as fixed variables, while for the PPH only UV‐B transmission was used as an explanatory variable. Models were compared using the same information‐theoretic approach as described for the spatial analysis and ranked according to AICc. As relative humidity and UV‐B values were assigned to assemblages within the subterranean stratum, we ran the analysis again omitting the subterranean stratum (Appendix S6).

## 3 RESULTS

**Figure 2:**
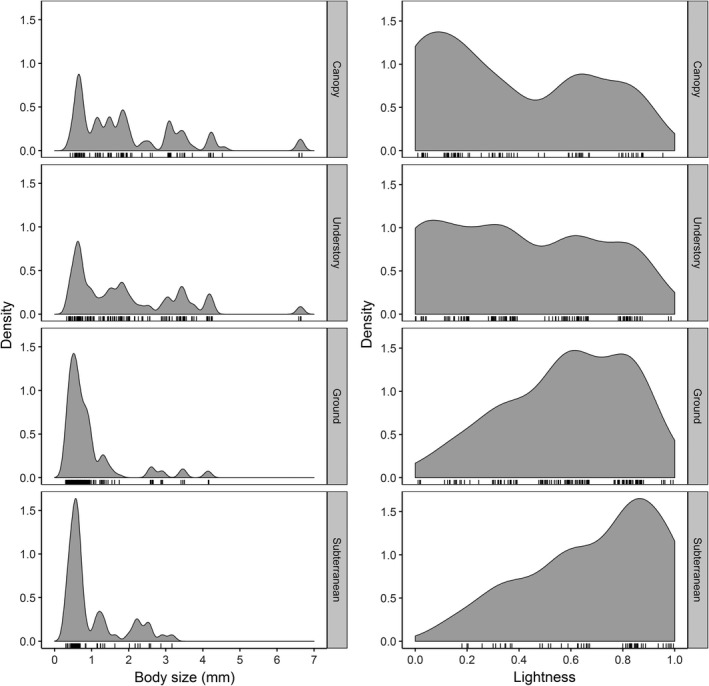
Density plots showing the distribution of body size (i.e. Weber's length) and lightness for species found within each stratum. Marks along the *x*‐axis indicate the actual distribution of species. The same bandwidth is used for body size and lightness

In total, 33,976 ant specimens were collected across four different strata, belonging to 231 morphospecies; of these morphospecies, colour and body size was recorded for 222. Species richness was highest in the understory stratum (*n* – 94 species) and lowest in the subterranean stratum (*n* – 37 species) (canopy, 62; ground, 83). Across all species, lightness values varied from 0 to 1 and body size varied from 0.29 mm to 6.63 mm. The distribution of lightness values and body size varied within each stratum with larger numbers of small and light species found in the ground and subterranean strata (Figure 2). Assemblage‐weighted lightness ranged from 0.20 to 0.83 and assemblage‐weighted body size from 0.67 mm to 1.60 mm.

There was no significant phylogenetic signal in lightness across the phylogenetic tree (Nelsen, Ree, & Moreau, 2018); lightness in closely related genera do not resemble each other more so than would be expected by chance (Pagel's *λ* = 0.430, *p* = .11; Blomberg's *K* = 0.803, *p* = .05). However, body size was conserved across the phylogeny, and a significant phylogenetic signal was identified (Pagel's *λ* = 0.612, *p* = .001; Blomberg's *K* = 0.929; *p* = .005). This signal can be accounted for as in the sampled assemblages genera in the subfamilies Ponerinae and Formicinae were larger than those in other subfamilies (Figure S4.2). However, proportional representation of both Ponerinae and Formicinae in the sampled assemblages do not correlate strongly with their assemblage‐weighted body size (Ponerinae, spearman's *ρ* = −0.25, *n* = 16, *p* = .35; Formicinae, spearman's *ρ* = 0.32, *n* = 16, *p* = .23); thus, we do not consider that the phylogenetic signal of body size confounds the analysis (Figure S4.4). A strong correlation between the proportions of an assemblage that are Ponerinae or Formicinae and assemblage‐weighted body size would have indicated that this phylogenetic signal was influencing the results.

### 3.1 Testing spatial patterns in cuticle colour

**Figure 3:**
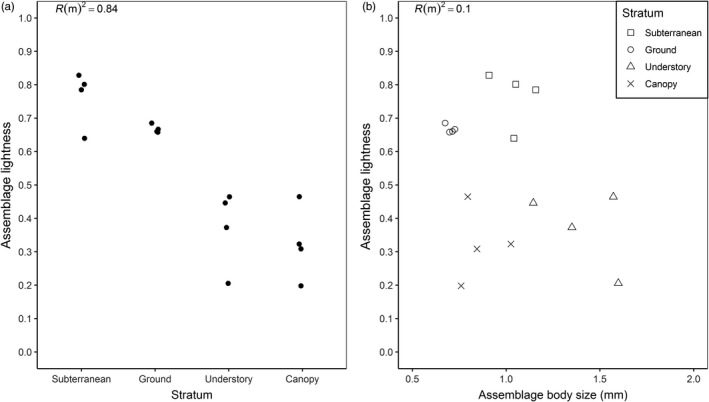
Plots showing the relationship between assemblage‐weighted lightness and (a) stratum and (b) assemblage‐weighted body size. Stratum is a significant predictor of lightness while body size is not (*n* = 16 assemblages; 4 plots × 4 strata)

The best descriptive model was also the simplest (see Table 1); stratum was the only significant predictor of assemblage‐weighted lightness (*F*_(3,12)_ = 26.91, *p* < .001). Marginal ($R_{m}^{2}$) and conditional ($R_{C}^{2}$) *r*‐squared values showed that almost all of the variance being described by the model was derived from the fixed term, stratum, with negligible variation explained by the random term plot ($R_{m}^{2}$ = 0.837, $R_{C}^{2}$ = 0.845). At the assemblage level, lightness declined with increasing height from the ground (Figure 3a). Pairwise comparisons showed that the understory and canopy assemblages were significantly darker than the ground and subterranean assemblages (see Appendix S6). Although there tended to be a higher density of smaller species found in lower strata, such as in the subterranean and ground layers (Figure 2), at the assemblage level, body size was not a significant predictor of lightness (Figure 3b). Species richness did not influence these results given that richness had no significant correlation with assemblage lightness (*t* = −0.96, *df* = 16, *p* = .351) and explained only a small amount of variation in assemblage lightness ($R_{m}^{2}$ = 0.06, $R_{C}^{2}$ = 0.06) (Appendix S4).

**Table 1:** Comparative statistics for linear mixed models explaining variation in ant assemblage lightness

Model	*df*	LL	AIC_c_	ΔAIC_c_	$R_{m}^{2}$	$R_{C}^{2}$
**~Stratum**	**6**	**−7.18**	**35.7**	**0**	**0.837**	**0.845**
~Stratum + AWM size	7	−6.61	41.2	5.52	0.849	0.849
~Intercept	3	−21.28	50.6	14.87	0	0
~AWM size	4	−20.47	52.6	16.89	0.102	0.102
~Stratum × AWM size	10	−6.12	76.2	40.55	0.858	0.858

Note

The response variable of assemblage‐weighted lightness, in all models, was logit transformed. Explanatory variables included fixed effects of stratum and assemblage‐weighted body size (AWM size); all models included a random effect of plot. Listed are the degrees of freedom (*df*), log‐likelihood (LL), bias‐corrected AIC (AIC_c_) and its change relative to the best descriptive model (ΔAIC_c_). Marginal *R* ^2^ ($R_{m}^{2}$) shows the amount of variation explained by the fixed effects while conditional *R* ^2^ ($R_{C}^{2}$) shows that explained by fixed and random effects. The most parsimonious model is highlighted in bold.

### 3.2 Testing hypotheses for patterns in cuticle colour

**Figure 4:**
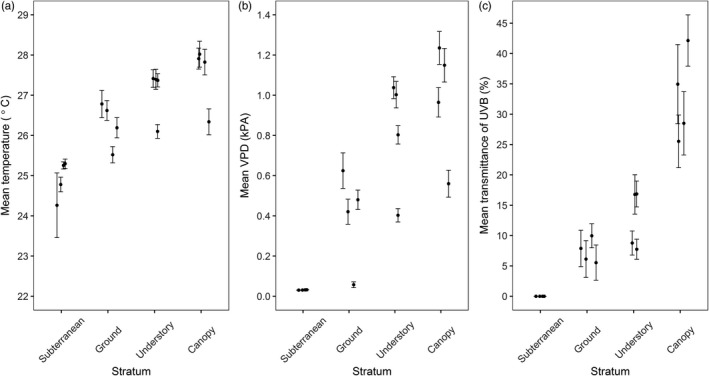
Dot plots showing the relationship between stratum and (a) mean temperature, (b) mean vapour pressure deficit (VPD) or (c) mean transmittance of UV‐B radiation, with 95% confidence intervals. Temperature refers to air temperature for ground, understory and canopy strata but to soil temperature for subterranean stratum. For subterranean stratum, VPD was calculated by assigning a humidity value of 99% while UV‐B transmittance was assigned as 0%. For each stratum: *n* = 4 plots

As expected, we found vertical gradients in temperature, VPD and UV‐B radiation. Mean temperature was highest in the canopy and lowest in the subterranean stratum (Figure 4a); with a difference in means of 2.6°C, this is equivalent to around 300–400 m of elevational change (on average, temperature drops by 0.6°C‐0.7°C with 100 m of elevational gain). Differences in maximum temperature were much larger with temperatures reaching 42.6°C in the canopy, 36.1°C at ground level and 26.2°C in subterranean stratum. VPD (the drying power of the air) increased with height (Figure 4b), with a mean VPD in subterranean and ground strata of 0.03 and 0.40 kPa, respectively, compared with 0.81 and 0.98 kPa in the understory and canopy strata. While transmission of UV‐B radiation also increased with height above ground (Figure 4c), mean transmittance of UV‐B in the canopy was 32.8% and only 7.4% at ground level.

**Figure 5:**
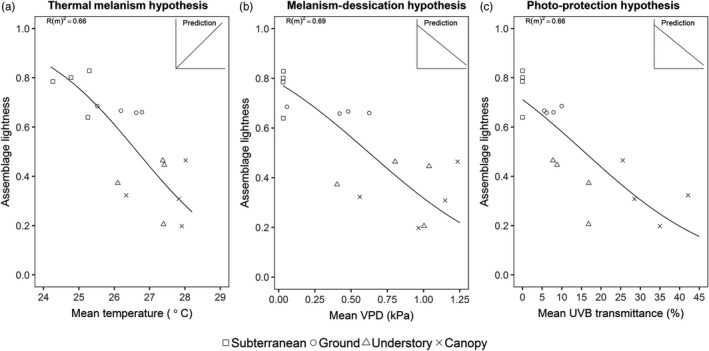
Relationship between assemblage‐weighted lightness and (a) mean temperature, (b) mean vapour pressure deficit (VPD) or c) mean UV‐B transmittance. Lines display model predictions and $R_{m}^{2}$ (fixed effects) are shown. For subterranean stratum, VPD was calculated by assigning a humidity value of 99% to each subterranean assemblage while UV‐B transmittance was assigned as 0% (*n* = 16 assemblages; 4 plots × 4 strata). Inset shows predicted relationship

The best model for explaining variation in assemblage lightness aligned with the melanism‐desiccation hypothesis and included only VPD as an explanatory variable (Table 2). However, delta AICc was less than three for the top five models, indicating that assemblage lightness could be explained by changes in all three abiotic variables (mean VPD, mean UV‐B and mean temperature). Assemblage lightness showed a negative relationship with VPD (Figure 5b) and with UV‐B (Figure 5c) as predicted by both the melanism‐desiccation hypothesis and the photo‐protection hypothesis (Figure 1). However, assemblage lightness also showed a negative relationship with temperature (Figure 5a) which is the opposite pattern predicted by the thermal melanism hypothesis. Similar patterns were evident if subterranean stratum was omitted from the analysis, with the best models including UV‐B or VPD as explanatory variables (Appendix S5).

**Table 2:** Comparative statistics for linear mixed models explaining variation in ant assemblage lightness according to each hypothesis

Hypothesis	Model	*df*	LL	AIC_c_	ΔAIC_c_	$R_{m}^{2}$	$R_{C}^{2}$
**MDH**	**~VPD**	**4**	**−11.78**	**35.2**	**0**	**0.692**	**0.854**
PPH	~UV‐B	4	−12.92	37.5	2.29	0.663	0.663
MDH	~VPD + AWM size	5	−10.77	37.5	2.34	0.721	0.869
TMH	~Temp	4	−13.19	38.0	2.82	0.661	0.759
TMH	~Temp + AWM size	5	−11.04	38.1	2.88	0.731	0.818
Null	~intercept	3	−21.28	50.6	15.37	0	0
TMH/MDH	~AWM size	4	−20.47	52.6	17.39	0.102	0.102

Note

The response variable of assemblage‐weighted lightness was logit transformed. Explanatory variables included fixed effects of mean vapour pressure deficit (VPD), mean ultraviolet‐B radiation (UV‐B), mean temperature (Temp) and assemblage‐weighted body size (AWM size). All models included a random effect of plot. For subterranean strata, VPD was calculated by assigning a humidity value of 99% while UV‐B transmittance was assigned as 0%. Listed are the degrees of freedom (*df*), log‐likelihood (LL), bias‐corrected AIC (AICc) and its change relative to the best descriptive model (ΔAICc). Marginal *R* ^2^ ($R_{m}^{2}$) shows the amount of variation explained by the fixed effects while conditional *R* ^2^ ($R_{C}^{2}$) shows that explained by fixed and random effects. The most parsimonious model is highlighted in bold.

Abbreviations: MDH, melanism‐desiccation; PPH, photo‐protection; TMH, thermal melanism.

## 4 DISCUSSION

Our study shows that patterns in cuticle colour can be detected at small spatial scales along microclimatic gradients. We found that cuticle colour was vertically stratified; canopy and understory ant assemblages were significantly darker than ground and subterranean assemblages. Our data support the MDH and the PPH, but not the TMH, as potential mechanisms driving this pattern in cuticle colour. Although patterns of colour in ectotherms have been extensively researched at the macroclimatic scale, across latitudes and elevations (Bishop et al., 2016; Zeuss et al., 2014), studies at the microclimatic scale are scarce (Chown & Gaston, 2016). Macroclimate is not always a good reflection of the habitat that an organism actually experiences (Kaspari et al., 2015; Stark et al., 2017), and there are growing calls to incorporate microclimatic data into analyses of trait–environment relationships and species distribution modelling (Chown & Gaston, 2016; Kaspari et al., 2015; Pincebourde & Casas, 2015).

The three main competing hypotheses that could explain the gradation in cuticle colour are as follows: thermal melanism, TMH, (Bogert, 1949), melanism‐desiccation, MDH, (Kalmus, 1941) and the photo‐protection, PPH. We dismiss the TMH as an explanation for the pattern we observe. The TMH predicts a positive relationship between cuticle lightness and temperature due to the thermoregulatory benefits of being light in warmer environments. However, our results show a strong negative relationship between cuticle lightness and temperature (Figure 5a). Vertical gradients in mean (range from 24.3°C to 28.0°C) and maximum temperature (range from 25.2°C to 42.6°C) should select for lighter colours in the canopy yet this is not what we observe. Greater thermal tolerances shown by canopy ants (Kaspari et al., 2015) may lessen the need for lighter cuticles despite the presence of higher temperatures. Rather than cuticle colour, ants may rely on other morphological and/or behavioural adaptations for thermoregulation (Kaspari et al., 2015; Stark et al., 2017; Sunday et al., 2014).

A competing hypothesis to explain the pattern we observe in cuticle colour is the MDH. This predicts that as VPD increases so does the risk of desiccation; thus, darker cuticles are advantageous due to reduced cuticular permeability (Kalmus, 1941). Our data support the MDH as we found a strong negative relationship between VPD and cuticle lightness (Table 2; Figure 5b). Experimental work on this topic has shown that tropical canopy ants are able to tolerate desiccation stress three times longer than ground ants but their ability to tolerate desiccation shows an inverse relationship with thermal tolerance (Bujan, Yanoviak, & Kaspari, 2016). This trade‐off between desiccation resistance and thermal tolerance has been attributed to cuticular permeability: more evaporative cooling through a permeable cuticle leads to greater thermal tolerance but reduced resistance to desiccation. If darker cuticle is less permeable then the darker assemblages, we observed in the canopy suggests that desiccation resistance exerts a greater selection pressure than thermal tolerance. However, a few studies have tested which levels of VPD lead to significant water loss in insects (Shipp & Van Houten, 1997). The mean VPD in the canopy of 0.98 kPa may not be high enough to act as a selection pressure on cuticle colour. Rather than the deposition of melanin, resistance to desiccation could be determined by other physiological factors such as differences in epicuticular lipids (Hood & Tschinkel, 1990).

Our findings can, however, also be interpreted in light of a third hypothesis: photo‐protection which predicts a decrease in cuticle lightness due to the protection increased melanization provides against UV‐B radiation. We found a strong vertical gradient in UV‐B radiation across our sampled strata with UV‐B radiation four times higher in the canopy than on the ground. Furthermore, we found a strong negative relationship between assemblage lightness and mean UV‐B transmittance (Figure 5c). The decrease in cuticle lightness could confer protection against this high UV‐B radiation. To our knowledge, the only other study that has directly measured variation in UV‐B radiation within a tropical rainforest found similar results, with only 2% of incident UV‐B radiation reaching the forest floor (Brown, Parker, & Posner, 1994) compared with 5%–10% in this study. In temperate forests, between 40% and70% of UV‐B radiation is absorbed within the canopy (Brown et al., 1994). We are the first to show that this is also true in tropical rainforest as between 58% and 75% of UV‐B radiation was lost within the canopy. Although the transmittance of UV‐B radiation to the forest floor can increase under disturbed canopies, in leafless sections and in canopy gaps (Brown et al., 1994; Yang, Miller, & Montgomery, 1993), the downward attenuation of UV‐B radiation within forests is both considerable and predictable and could select for the patterns in cuticle colour we observe. Furthermore, experimental studies have demonstrated the physiological benefits that darker cuticles confer with regards to UV‐B protection (Mosse & Lyakh, 1994; Wang et al., 2008). Although the UV‐B model was not the most parsimonious model, delta AICc was less than three indicating substantial support for UV‐B as a mechanism explaining patterns of assemblage lightness (Burnham & Anderson, 2002) (Table 2).

So which mechanism, the MDH or the PPH, is driving darker cuticles in the canopy? The collinearity of VPD and UV‐B radiation in this study system does not allow us to easily disentangle the two; our data support both. We recognize that components of the abiotic environment often correlate and both VPD and UV‐B may influence the same trait.

The VPD and humidity values we record from the canopy stratum, a ‘dry’ environment in our context, are comparable to high humidity and ‘wet’ conditions in other studies investigating the MDH (Parkash et al., 2008, 2009). As a result, it is reasonable to question whether VPD is high enough in the canopy, or the difference between strata pronounced enough, to select for differences in cuticle colour. Our data may be more congruent with studies that have addressed photo‐protection in ectotherms, and particularly so in tropical regions. Environmental factors that shape trait variation are likely to differ between temperate and tropical regions. In temperate regions, darker cuticles of *Drosophila* at higher latitudes have been linked to the thermoregulatory benefits of melanism (David, Capy, Payant, & Tsakas, 1985; Parkash & Munjal, 1999), while in tropical regions darker cuticles of *Drosophila* are found at lower latitudes and attributed to greater UV‐B radiation (Bastide et al., 2014). A similar discrepancy has been found in ants where across numerous elevational gradients cuticle lightness of ant assemblages increased with temperature; however, this did not hold true in environments where temperature increased beyond 24–25°C (Bishop et al., 2016). The mean temperature of the coolest stratum in this study falls within this high temperature range supporting the suggestion made by Bishop et al. (2016) that other selective pressures, primarily high UV‐B radiation, take precedent in driving cuticle colour in hot environments.

Further work in systems where VPD and UV‐B are not correlated would be helpful in disentangling the MDH from the PPH but are perhaps difficult to find. Rather, laboratory experiments may provide further insights into the physiological benefits of melanization under various abiotic conditions. For example, when holding body size constant, does melanization of the cuticle result in a reduction in water loss? At what point does VPD affect fitness? And, does higher melanization slow or minimize damage to cells and organs associated with UV‐B?

Further caveats include the aggregation of nocturnal and diurnal species within our dataset. For species that forage solely at night, it may be expected that cuticle colour is decoupled from the influence of temperature and UV‐B radiation while desiccation gradients may still exist. While in the tropics nocturnal and diurnal ant communities can differ, few species are exclusively nocturnal (Houadria, Salas‐Lopez, Blüthgen, Orivel, & Menzel, 2015). Further studies are needed to determine the effects of circadian asynchrony in tropical ant communities. Finally, we acknowledge that melanin has been shown to enhance immunity in insects (Wittkopp & Beldade, 2009), and thus, the prevalence of parasites and pathogens may also select for darker cuticles. However, to our knowledge, there are no data on the spatial variation of ant parasites and pathogens within tropical forests.

The patterns in cuticle we detected were at the assemblage level and therefore reflect changes in the relative abundance of species. Using an assemblage average best captures the performance of different lightness values among strata and under different microclimates. We expect that changes to the abiotic environment will alter optimal cuticle lightness and lead to a shift in assemblage structure. Changes in assemblage lightness over an ecological time frame have been demonstrated in montane ant assemblages whereby assemblage lightness changed over a period of years as the environment fluctuated (Bishop et al., 2016). Considering the continued threat of deforestation in tropical regions (Hansen et al., 2013), existing microclimatic gradients in tropical rainforests are vulnerable to change. Removal of trees and alterations to canopy structure can render the understory, hotter, lighter and subject to greater solar radiation (Brown, 1993; Carlson & Groot, 1997). Under such change, we may expect darker ants to have a selective advantage due to the protective role of their darker cuticles. Existing literature suggests that darker, melanic individuals will be favoured under future climate warming due to an increase in UV‐B radiation (see review by Roulin, 2014). However, predicting optimal cuticle lightness under changing microclimates in tropical ecosystems is likely to depend on the relative importance of temperature, VPD and UV‐B radiation to the organism in question.

In summary, this is the first study to show evidence for the vertical stratification of cuticle lightness in tropical ant assemblages. We add to the small number of studies that examine ecogeographical rules at the microclimatic scale and at a level that more suitably describes the heterogeneous environment experienced by small ectotherms. We show that ant assemblages are darker, on average, in the canopy and lighter beneath the ground. This can be interpreted in light of the melanism‐desiccation hypothesis or the photo‐protection hypothesis but not the thermal melanism hypothesis. Continued alterations to forest structure and the subsequent changes to microclimate could result in changes to the relative abundance of particular ant species.

## AUTHORS' CONTRIBUTIONS

C.P. conceived the idea; S.J.L. designed the study, performed research, analysed data and wrote the paper; T.R.B. contributed to data analysis and critically to drafts; H.G. and L.A. contributed to fieldwork; P.E. and C.P. designed the study, contributed to data collection and contributed critically to drafts; all authors gave final approval for publication.

## Data Availability

Data are archived with the Environmental Information Data Centre (EIDC)/NERC, https://doi.org/10.5285/01034680-e640-44a2-aab6-2044b4672a95 (Law et al., 2018) and https://doi.org/10.5285/c0f65bf5-3cb0-41ab-be3d-982490cecba9 (Law et al., 2019).
