# Supplementary material for: Darker ants dominate the canopy: Testing macroecological hypotheses for patterns in colour along a microclimatic gradient
Source: J Anim Ecol. 2019 Oct 21;89(2):347–59. doi: 10.1111/1365-2656.13110 (PMC7027836; doi:10.1111/1365-2656.13110)
Supplement: Supplementary file 1 [file JANE-89-347-s001.docx]

SUPPORTING INFORMATION

Appendix S1 – Data collection

S1.1 Ant collection

Subterranean ants were collected from February to May 2016 using baited traps. Traps were capped plastic vials (50 ml), 12 cm high and 2.5 cm in diameter, with four holes (0.5 cm in diameter) drilled in the upper half to allow access by ants. To attract ants, a mixture of honey and tuna was smeared on the inner walls above the access holes using a fine paint brush. Ethanol (70%) was placed below the access holes to retain and preserve ants. A uniform hole was made in the soil for each trap using a hand-held augur to prevent alterations to surrounding soil structure, each trap was placed in a hole and covered with soil. Traps were buried at a depth of 24 cm; similar traps and depths have been used to capture cryptobiotic species (Andersen & Brault, 2010; Pacheco & Vasconcelos, 2012; Ryder Wilkie, Mertl, & Traniello, 2007; Schmidt & Diehl, 2008; Yamaguchi & Hasegawa, 1996). The depth of traps was measured as the distance between the soil surface and the bottom of the trap. The efficiency of subterranean traps increases with extended periods of time (Andersen & Brault, 2010; Pacheco & Vasconcelos, 2012) thus traps were left for seven days. A length of string extending from the cap of each trap indicated the location and aided retrieval. To prevent traps being dug up by forest pigs a sheet of metal was pegged over the top of each trap.

Ground-dwelling, leaf litter, ants were collected during June and July 2014 using Winkler bag extractions. Ants were identified from seven Winkler bag extractions per plot, collected along two 50 m transects running diagonally from opposite corners. For each extraction 1 m^2^ of leaf litter was collected from the forest floor and sieved; the sieved material was transported to the lab in cotton bags and suspended in Winkler bags for 3 days.

Arboreal ants were collected from February to May 2016 using baited traps similar to the baited pitfall trap method described by Yusah, Fayle, Harris and Foster (2012). Altogether 12 trees were selected for our study: three trees within each plot that were either emergent or reached the high canopy. It was not possible to select trees of the same species due to the limited number of trees safe to climb within each 50 m x 50 m plot, however all trees were Dipterocarpaceae belonging to the genera *Parashorea* or *Shorea*. All trees were measured with a tape measure to the highest climbable point and the total height then estimated to the nearest meter. The surveyed trees ranged in total height from 27 to 52 m (mean $\pm$SD = 38 m $\pm$ 8) with the diameter at breast height (dbh) ranging from 41 to 105 cm (mean $\pm$SD = 66 cm $\pm$ 20).

Traps were placed at 5 m vertical intervals from the ground to as high into the canopy as possible within each tree. At each sampling station, traps comprised two plastic cups 8 cm in diameter tied together with a piece of string. In one cup a carbohydrate bait was placed (oat and honey mixture) and in the second cup a protein (tuna) bait. Baits were suspended in mesh bags hung from the centre of a wire across the top of each cup. Water containing a small amount of detergent was placed in the bottom of cups to break the surface tension. The stratum (canopy or understory) of each trap location was also recorded, traps placed above the first branch were identified as the canopy and traps below the first branch as the understory. Pairs of traps were hung on opposite sides of the trunk at each height interval. Traps were hung over nails to the tree trunk separating the cups within each pair by 20 cm and left open for 24 hours. In total 144 traps were set in the canopy and 272 in the understory across four plots. The canopy was accessed using the single rope technique and all climbers were attached to an additional safety line. Each tree was sampled once in all plots.

S1.2 Abiotic data

Estimates of air temperature and relative humidity were recorded for ground, understory and canopy strata during February to May 2016. Climate measurements were recorded by placing data loggers (Thermocron ibuttons®, model DS1923) at 5 m vertical intervals from the ground to the canopy on the largest sampled tree within each plot. At each height data loggers were hung within a plastic cup to protect them from rain, air vents were cut into the side of each cup and left continuously for five days. Values for temperature and humidity were recorded by the data loggers every 30 minutes. These values for temperature and humidity were used to calculate the vapour pressure deficit (VPD). Mean climatic values (and standard error) for each stratum were calculated using only the data loggers placed within the range of each stratum (i.e. only data loggers placed above the first branch were used to calculate mean temperature for the canopy). Soil temperature was recorded for subterranean assemblages during October 2016 using a digital temperature probe at an approximate depth of 10 cm. Soil temperature was recorded at 25 points within each plot, points were spread evenly across a grid and separated by 5 m. Measurements for soil temperature were taken on a single day in each plot. These soil temperature measurements were used with a humidity value of 99% to calculate values of VPD.

UV-B radiation was measured during October-November 2018, on the same trees and at the same 5 m vertical intervals as temperature and humidity. Although UV-B radiation was recorded at a different date to ant collection, seasonal variation in UV-B radiation at low latitudes is minimal (Beckmann et al., 2014; Appendix S3). All readings were taken within one hour of solar noon using a Solarmeter® (Model 6.0) that responds to wavelengths between 235 and 330 nm. Although these wavelengths are within the lower range of UV-A (315 – 400 nm) and upper range of UV-C (100 – 280 nm), the Solarmeter® is most sensitive to wavelengths within the UV-B range (280 – 315 nm) as such we refer to only UV-B radiation. At each height, 12 measurements were taken at arm’s length at approximately 10 second intervals over a 180^o^ angle and a mean recorded for each height. Mean UV-B transmittance for each stratum was calculated using measurements taken within the range of each stratum. Additionally, UV-B was recorded at 12 random points on the ground within each plot to estimate UV-B at ground level and a further 12 readings were taken in a clear, open area with no obstructions to the sky; the latter was used as a proxy for incident radiation on top of the canopy. Transmittance of UV-B radiation was defined as the irradiance recorded (at a specific height) divided by the incident radiation (clear sky). All measurements were only taken if there was less than 50% cloud cover. It was assumed that transmittance of UV-B radiation to the subterranean stratum was zero.

Appendix S2 – Colour assignment and trait variability

To test for observational error in assigning colour, a standardized set of 71 photographs from AntWeb (<http://antweb.org/>) was sent to 16 observers to assign colour to the head, mesosoma and gaster using the same colour wheel used in this study (Fig. S2.1). Error was low (Fig. S2.2), with the standard error of lightness values estimated from the 17 different observers (16 observers plus the author) on the same photograph averaging 0.026 for the head, 0.023 for the mesosoma and 0.026 for the gaster. The authors estimate for lightness strongly correlated with the mean estimate for lightness taken from the 16 observers for all body parts (Fig. S2.3). Data for testing this method came from Bishop et al. (2016).

To test for intraspecific variation, colour and body size was recorded for 20 species using a larger sample size. For each species, a single observer (the author, SL) assigned colour to the head, mesosoma and gaster and measured body size (i.e. Weber’s length) for 50 specimens. Species were selected based on the following criteria: 1) species with at least 50 specimens, 2) to include species found in a single stratum and species found across strata, 3) to cover a wide range of size and lightness values, and 4) to sample a range of genera and subfamilies. Only 39 of the 222 species recorded had an abundance of 50 or more, from which 20 species were selected. Of these 20 species: 10 were found in a single stratum while 10 were found across two strata, mean body size ranged from 0.29 to 4.27 mm and mean lightness values ranged from 0 to 0.83, and species covered 17 genera and 5 subfamilies. Intraspecific variation was low for both body size and lightness values; the mean coefficient of variation of body size values estimated for 20 species was 0.053 while the mean coefficient of variation of lightness values estimated for 20 species was 0.054 for the head, 0.089 for the mesosoma and 0.115 for the gaster (Fig. S2.4).


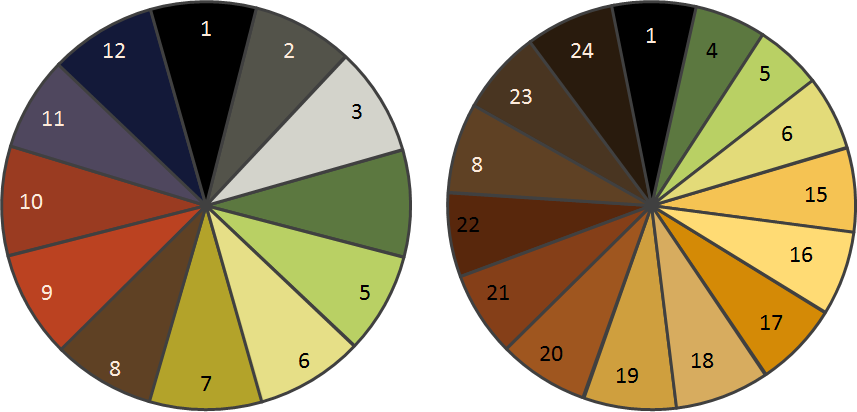


Figure S2.1: Colour wheels used to assign colour to the head, mesosoma and gaster of ants collected across all strata (subterranean, ground, understory and canopy).


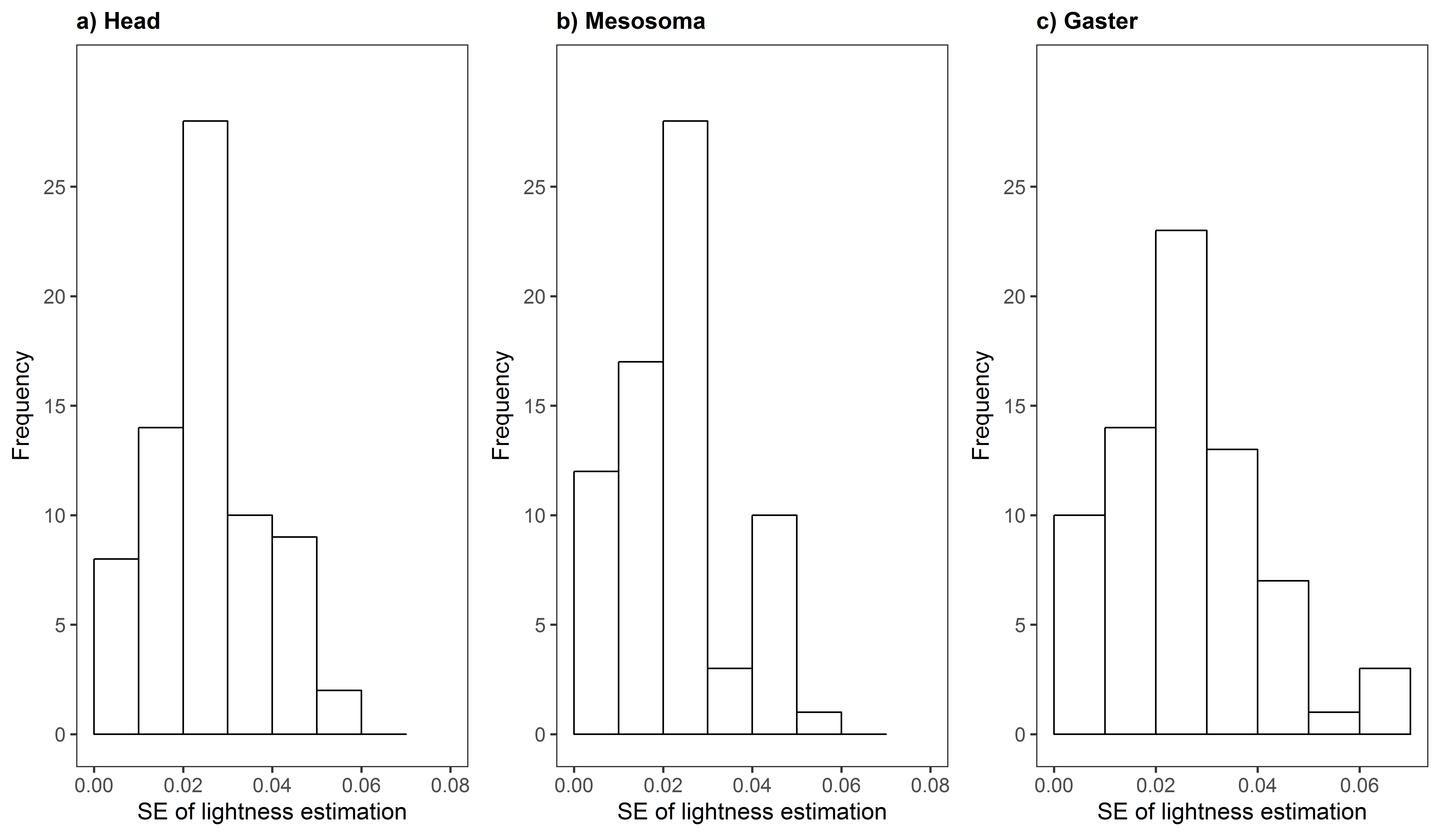


Figure S2.2: Histograms showing the standard errors of lightness values estimated for the a) head, b) mesosoma and c) gaster by 17 observers, including the author, on a set of 71 photographs from antweb.org. Mean standard error for the head is 0.026, mesosoma is 0.023 and gaster is 0.026.


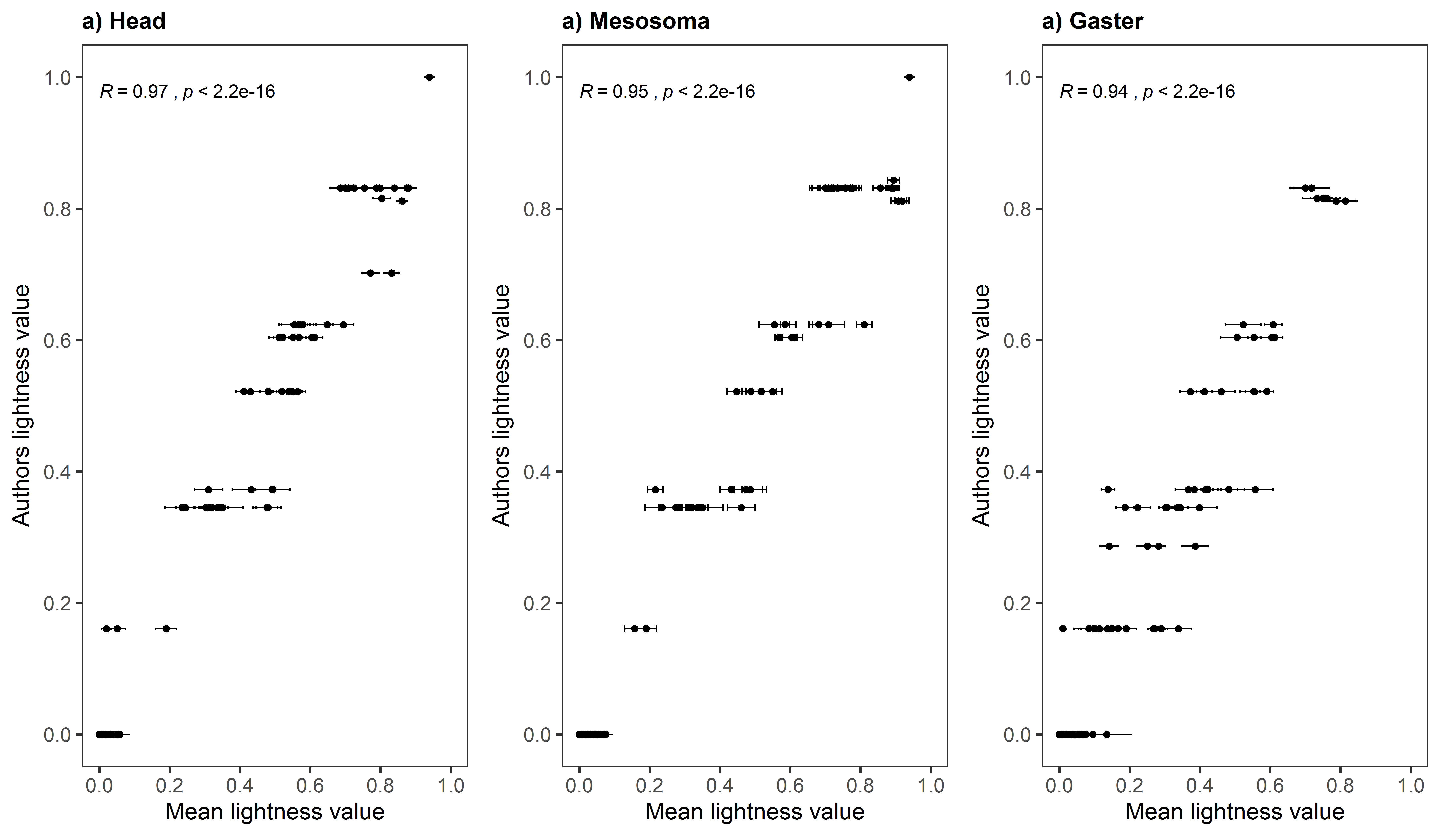


Figure S2.3: Relationship between the lightness value assigned by the author and the mean lightness values estimated from 16 observers for the a) head, b) mesosoma and c) gaster on a set of 71 photographs from antweb.org. Spearman’s correlation results are shown.





Figure S2.4: Coefficient of variation of lightness values estimated for the a) head, b) mesosoma and c) gaster and d) of body size values for 20 species of ants. Each point represents a species of which lightness and body size (i.e. Weber’s length) was measured for 50 specimens. Mean coefficient of variation for the head is 0.054, mesosoma is 0.089, gaster is 0.115 and body size is 0.053.


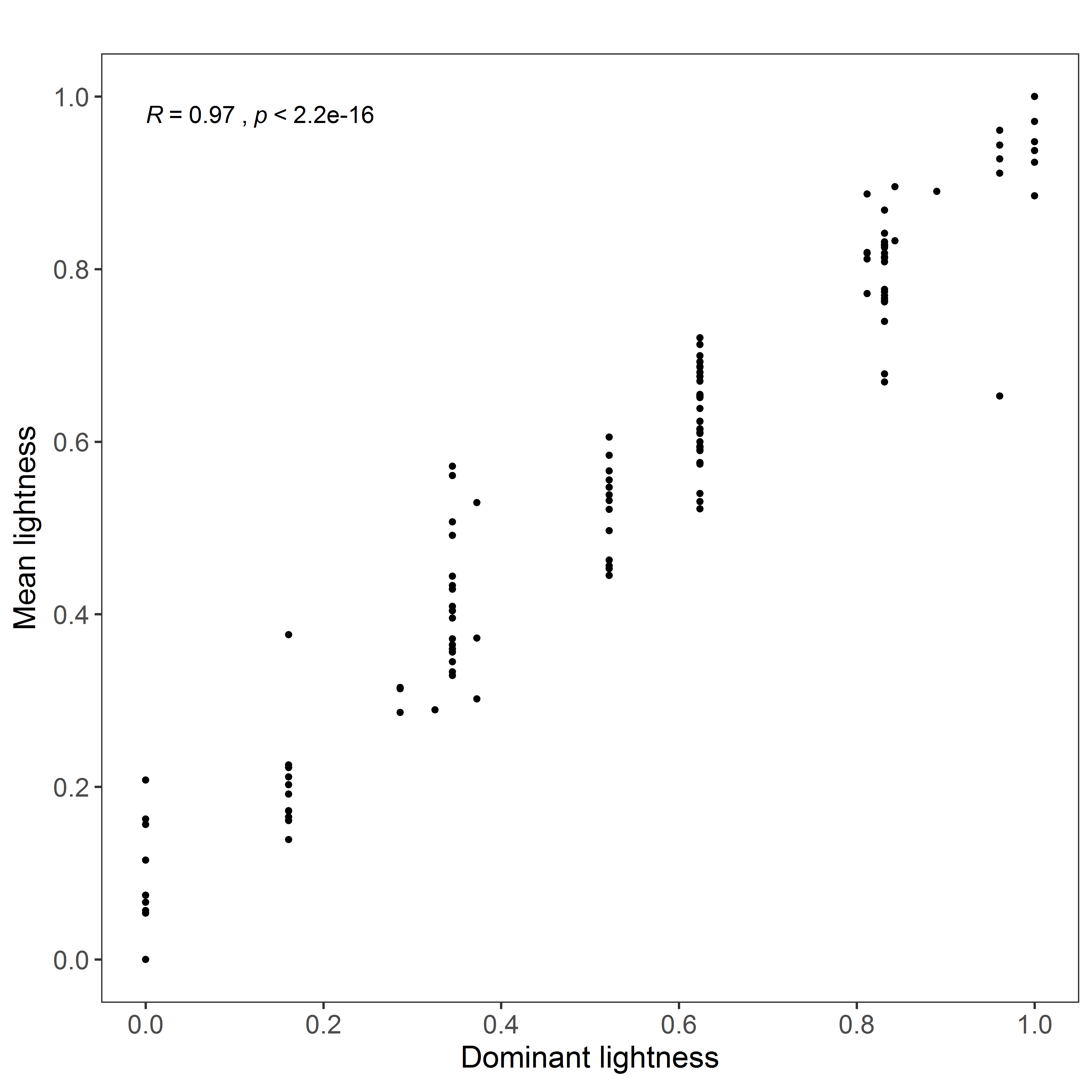


Figure S2.5: Relationship between dominant lightness and mean lightness values for each species (n = 222). Dominant lightness is the most common colour across all body parts and individuals for each species. Mean lightness is the mean lightness across all body parts and individuals for each species. Spearman’s correlation results are shown.

Appendix S3 – Variation in UV-B radiation

Levels of UV-B radiation were extracted from the glUV dataset (Beckmann et al., 2014). We compared the twelve monthly mean UV-B values (Jan – Dec) for our study site Maliau, which is at a low latitude (4° 44' 35 N and 116° 58' 10" E), with a high latitude location, Liverpool (53° 24' 36 N and 2° 58' 48" W). Each monthly mean value is averaged over a 9 year period from 2004 to 2013. We also compared annual UV-B seasonality for each location, this is calculated as the standard deviation of mean monthly values. Monthly mean UV-B radiation was higher (Fig. S3.1) and less variable (Fig. S3.2, S3.3) at our low latitude study site, Maliau, compared with higher latitudes.


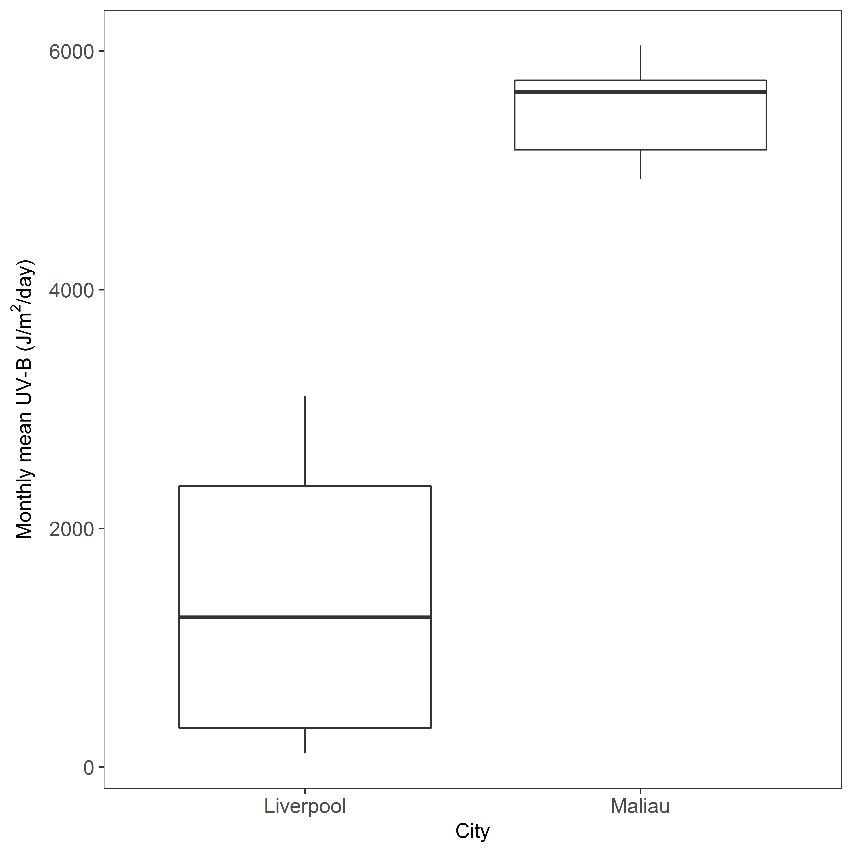


Figure S3.1: Box-plots showing differences between monthly mean UV-B radiation for a high latitude location, Liverpool, UK (53° 24' 36 N and 2° 58' 48" W) and a low latitude location, Maliau, Sabah, Borneo (4° 44' 35 N and 116° 58' 10" E), n = 12 (months, Jan – Dec).


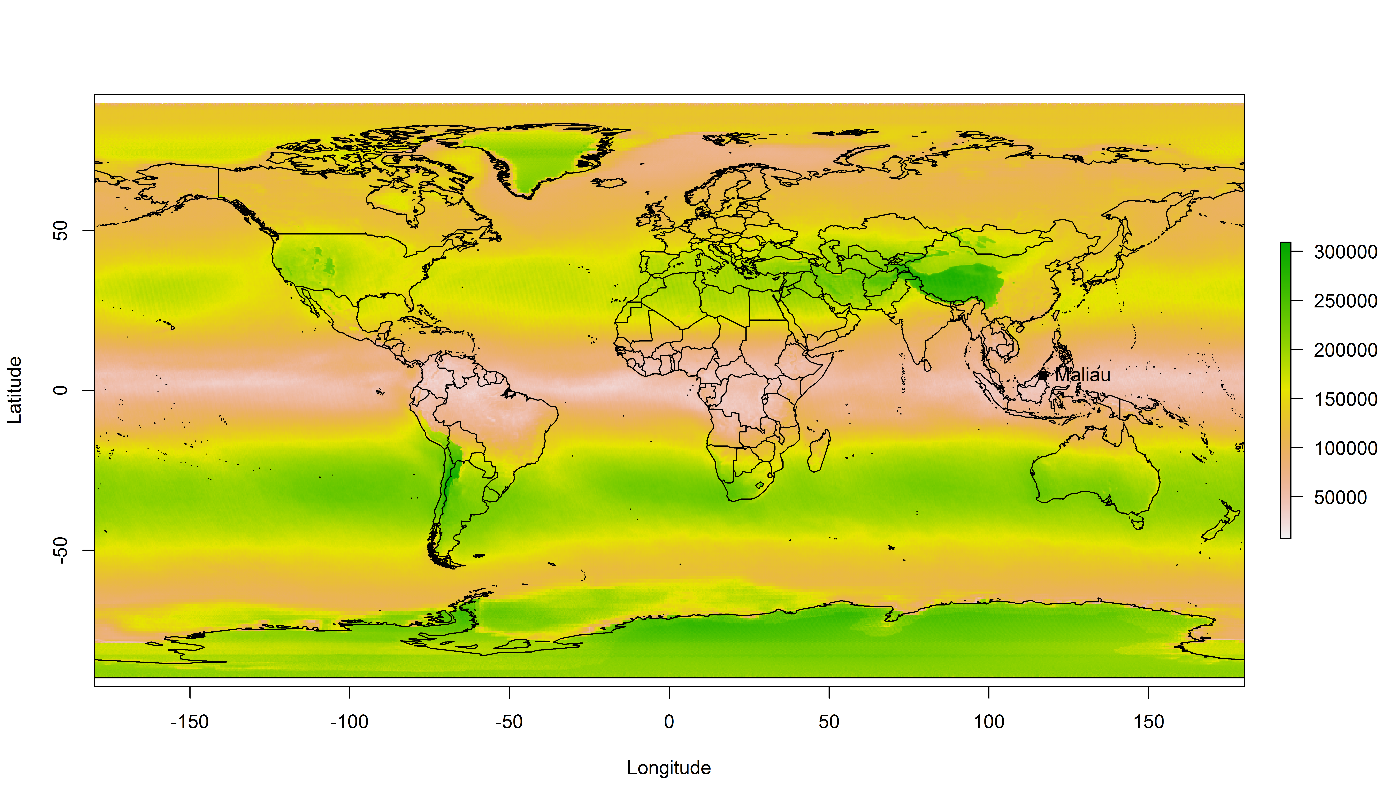
Figure S3.2: Map shows annual UV-B seasonality across the world. Seasonality is calculated as the standard deviation of monthly means. Indicated is our study site, Maliau, Sabah, Borneo, located in a region of very low seasonality.


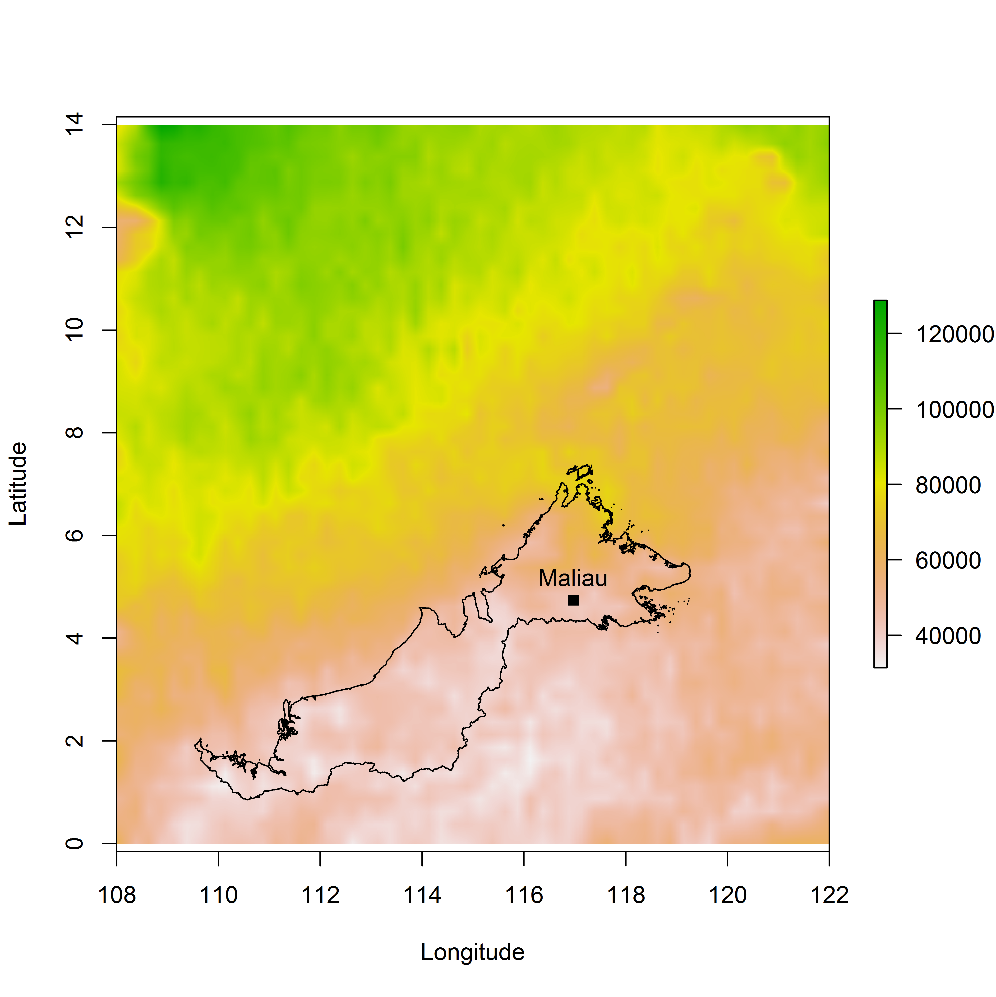


Figure S3.3: Higher resolution map showing annual UV-B seasonality across Malaysian Borneo, indicated is our study site Maliau. Seasonality is calculated as the standard deviation of monthly means.

Appendix S4 – Phylogenetic signal

To test whether variability in lightness and body size was linked to phylogeny, phylogenetic signal was estimated with Pagel’s λ (Pagel, 1999) and Blomberg’s K (Blomberg, Garland, & Ives, 2003) using a genus-level, time calibrated phylogeny derived from Nelsen, Ree and Moreau (2018). In this study 90% of the genera were present on the phylogeny. Missing genera (n = 5) were grafted on to the phylogeny adjacent to the closest related genus by the addition of new tip labels (Fig. S4.1). Existing literature was used to identify the closest related genus for missing genera (Blaimer, Ward, Schultz, Fisher, & Brady, 2018; Ward, Blaimer, & Fisher, 2016; Moreau & Bell, 2013). Phylogenetic signal was tested using the derived phylogeny with all genera and with missing genera omitted from the analysis. Lightness and body size traits were averaged at the genus level to test for signal. Using the ‘phylosig’ function from the package ‘phytools’ in R (Revell, 2012), a likelihood ratio test was used to assess if there was a significant departure of these statistics from zero (no phylogenetic signal). Significance of test statistics were not altered by omitting the missing genera, we report statistics using the derived phylogeny with all genera present.


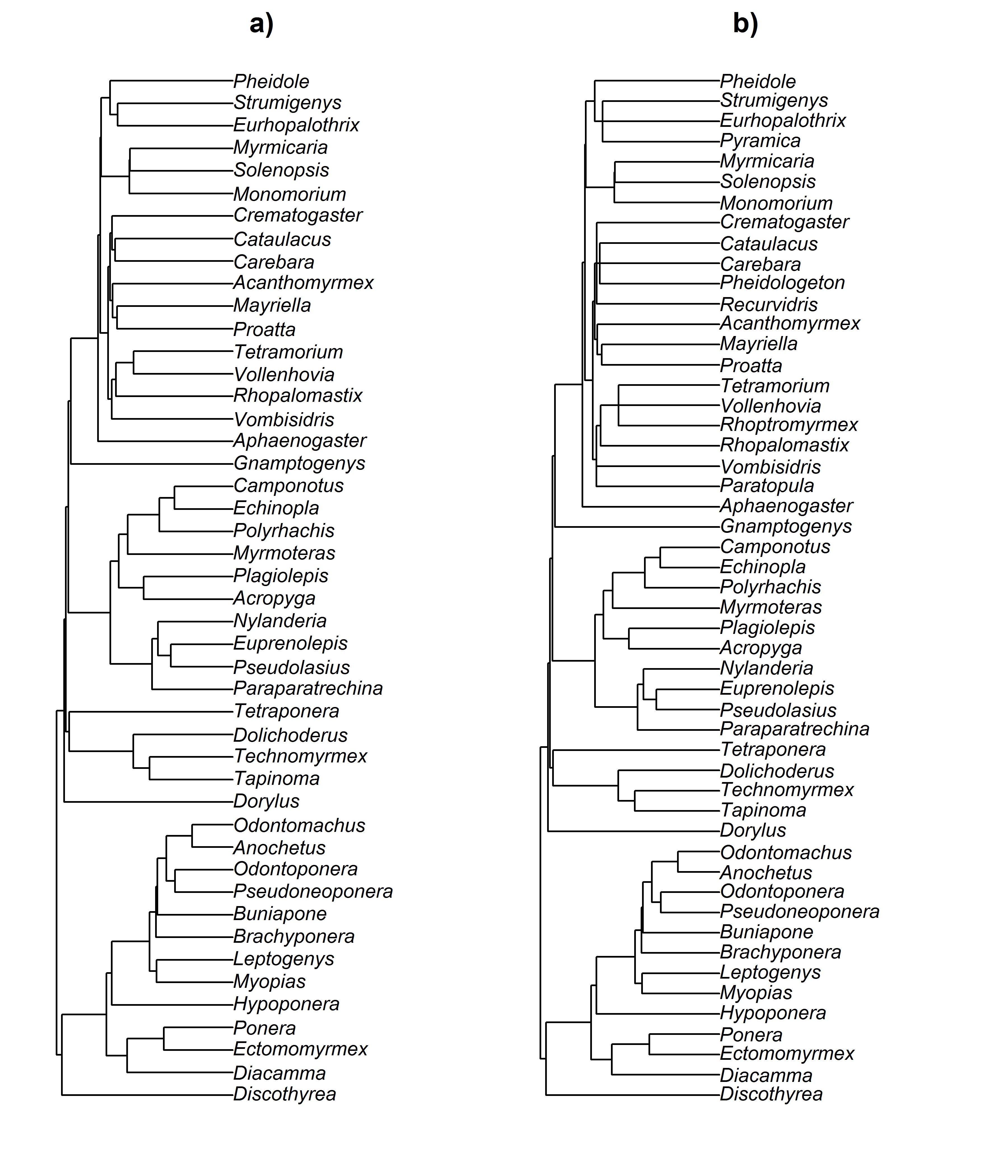
Figure S4.1: Time-calibrated phylogenies derived from Nelsen et al. (2018): a) genera present in both the study and original phylogeny (n = 46) and b) all genera present in the study (n = 51). In b), genera missing from the original phylogeny are grafted on adjacent to the most closely related genus identified from existing literature (Blaimer et al., 2018; Ward et al., 2016; Moreau & Bell, 2013)*.* Genera grafted on include: *Paratopula, Pheidologeton, Pyramica, Recurvidris* and *Rhoptromyrmex.*


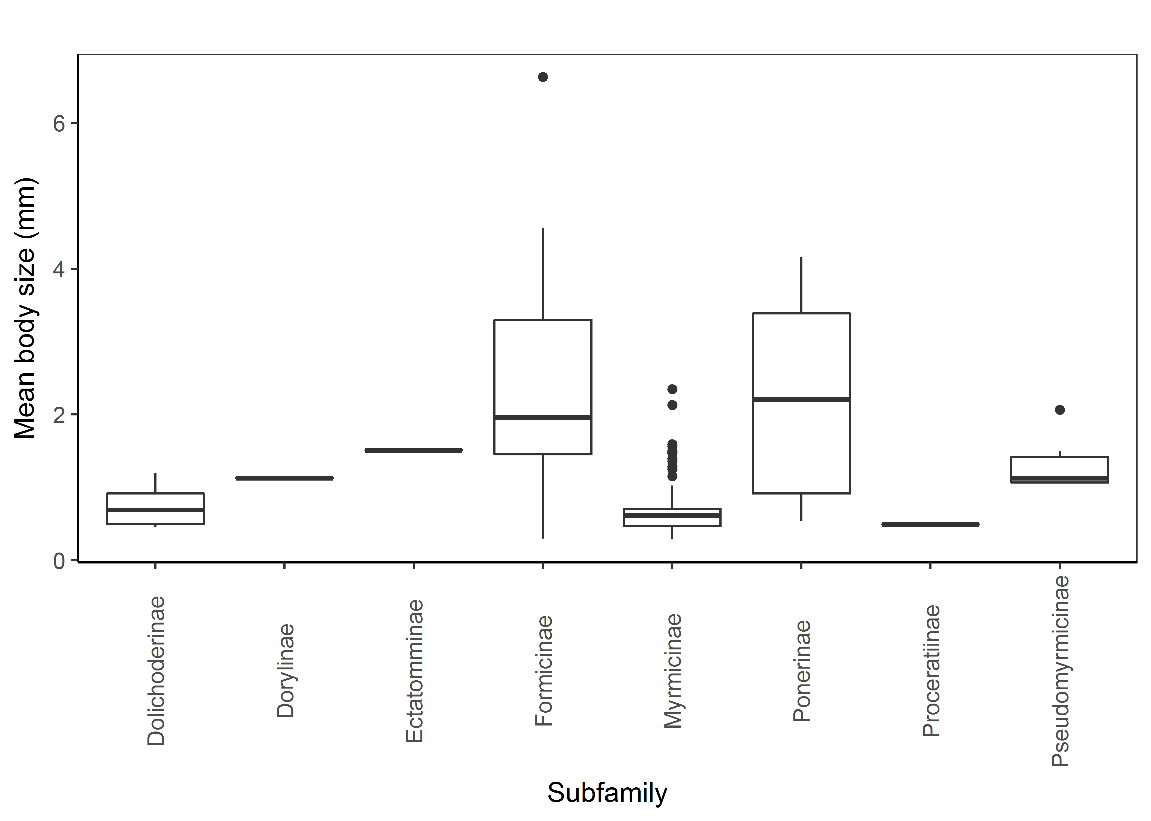


Figure S4.2: Plot showing the distribution of mean body size across the ant subfamilies present in this study.


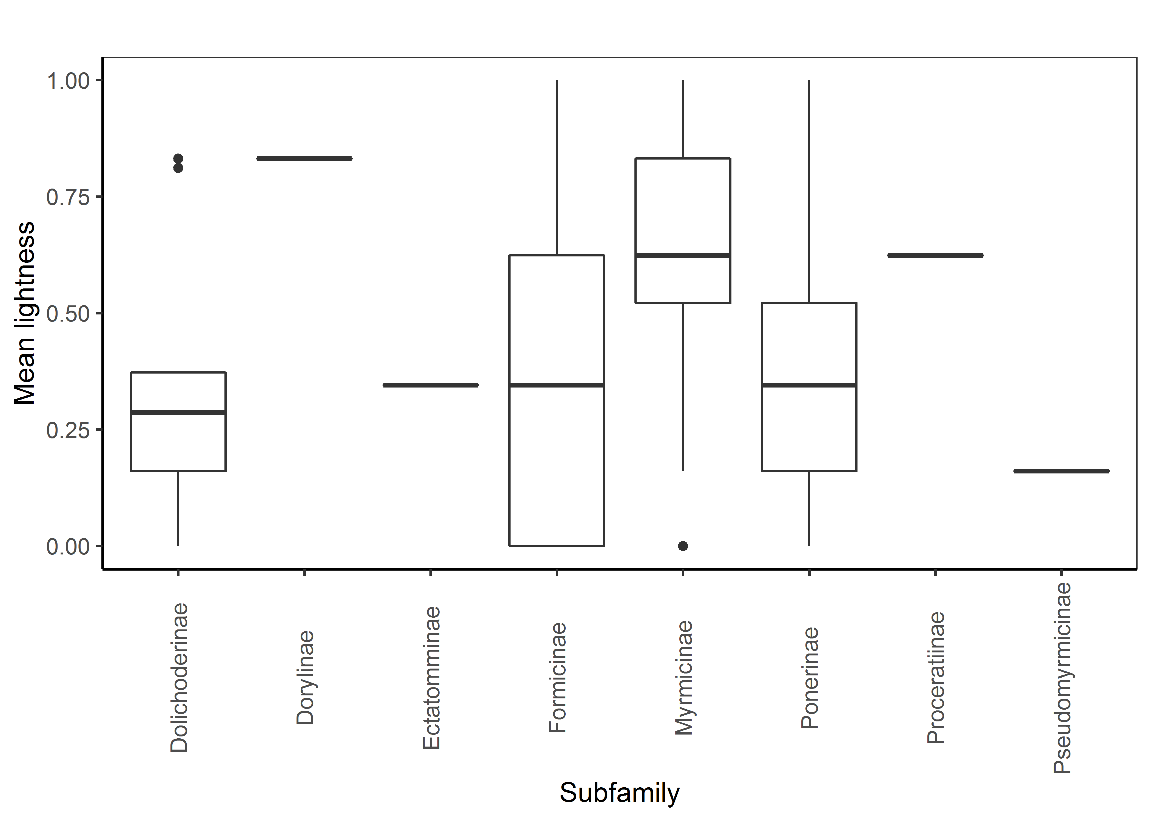


Figure S4.3: Plot showing the distribution of lightness across the ant subfamilies present in this study.


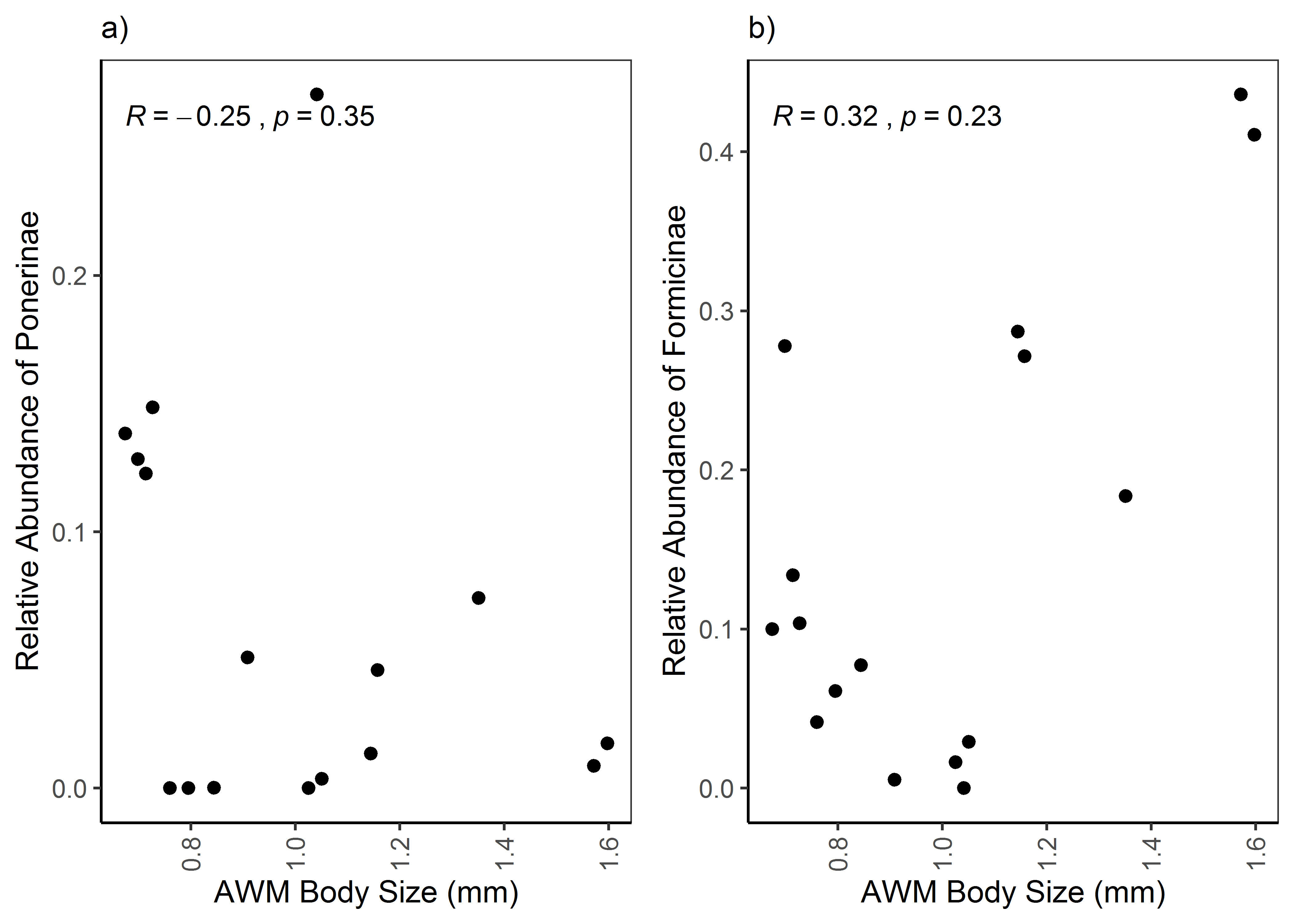


Figure S4.4: Relationship between the proportional representation of a) Ponerinae and of b) Formicinae in the sampled assemblages and the assemblage weighted mean (AWM) for body size (n = 16 assemblages; 4 plots X 4 strata).

Appendix S5 – Species richness effects


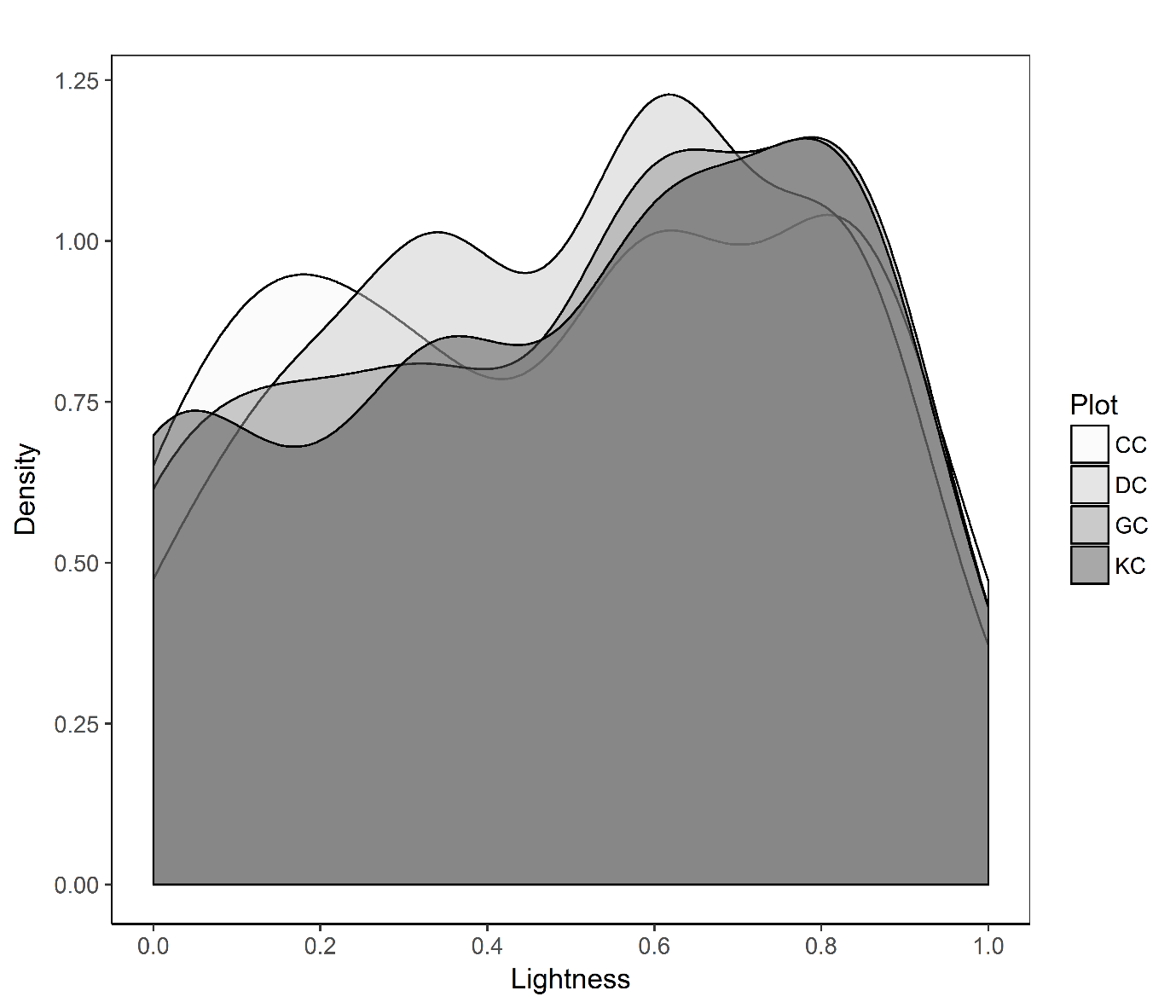


Figure S5.1: Stacked density plot showing the distribution of lightness values for each plot. Underlying data is at the morphospecies level. Number of species in each plot are as follow: CC (n = 105), DC (n = 84), GC (n = 90), KC (n = 95).


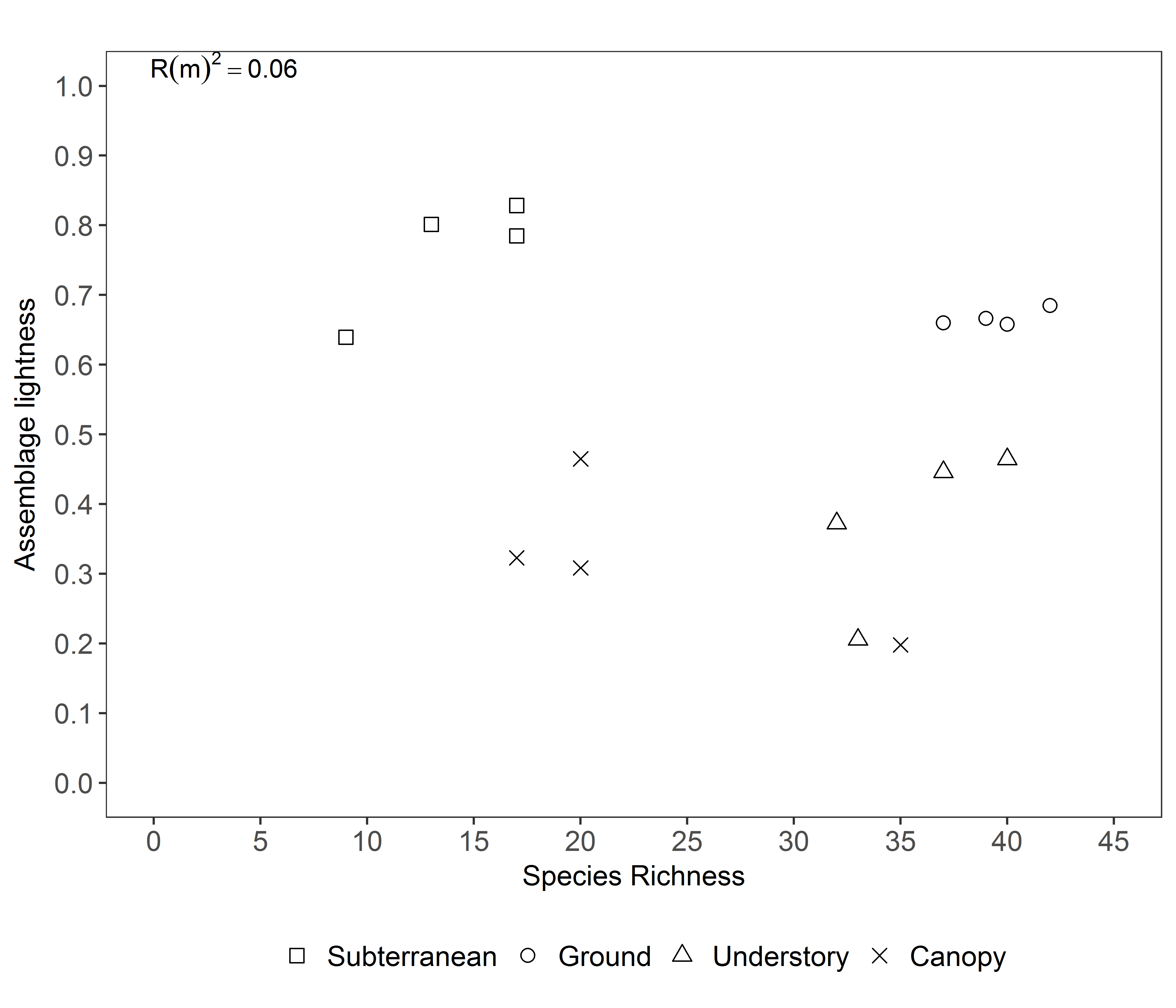
Figure S5.2: Relationship between assemblage weighted lightness and species richness (n = 16 assemblages; 4 plots X 4 strata).

Appendix S6 – Testing hypotheses for patterns in cuticle colour

Table S6.1: Test statistics from the best descriptive linear mixed model. The response variable, assemblage weighted lightness, was logit transformed. The fixed effect, stratum, included the following levels: subterranean, ground, canopy and understory. Listed are mean squares (Mean Sq.), numerator degrees of freedom (Num d.f.) and denominator degrees of freedom (Den d.f.) and p-values calculated from Type 3 F-statistics with Satterthwaite approximation for degrees of freedom. Post-hoc pairwise comparisons were calculated based on differences of least square means using the ’difflsmeans’ function in the package lmerTest.

| ANOVA | | | | | |
| --- | --- | --- | --- | --- | --- |
| Fixed effects | Mean Sq. | Num d.f. | Den d.f. | F | Pr (>F) |
| ~ stratum | 3.697 | 3 | 12 | 26.914 | <0.001 *** |
| Pairwise comparisons using difflsmeans | | | | | |
|  | Estimate | SE | d.f. | t | Pr (>\| t \|) |
| Subterranean ~ Ground | 0.512 | 0.262 | 12 | 1.955 | 0.074 |
| Subterranean ~ Understory | 1.767 | 0.262 | 12 | 6.743 | <0.001 *** |
| Subterranean ~ Canopy | 1.981 | 0.262 | 12 | 7.561 | <0.001 *** |
| Ground ~ Understory | 1.255 | 0.262 | 12 | 4.788 | <0.001 *** |
| Ground~ Canopy | 1.469 | 0.262 | 12 | 5.606 | <0.001 *** |
| Understory ~ Canopy | 0.214 | 0.262 | 12 | 0.818 | 0.429 |

Table S6.2: Comparative and summary statistics for linear mixed models explaining variation in ant assemblage lightness according to each hypothesis (TMH – thermal-melanism; MDH – melanism-desiccation; PPH – photo-protection). The response variable of assemblage weighted lightness, in all linear mixed models, was logit transformed. Explanatory variables included fixed effects of mean vapour pressure deficit (VPD), mean ultraviolet-B radiation (UVB), mean temperature (temp) and assemblage weighted body size (AWM size). All linear mixed models included a random effect of plot. Listed are the degrees of freedom (d.f.), log-likelihood (LL), bias corrected AIC (AICc) and its change relative to the best descriptive model (ΔAICc). Marginal R^2^ (R^2^m) shows the amount of variation explained by the fixed effects while conditional R^2^ (R^2^c) shows that explained by fixed and random effects. The most parsimonious model is highlighted in bold. Subterranean strata were omitted from the analysis.

| Hypothesis | Model | d.f. | LL | AIC_c_ | ΔAIC_c_ | R^2^_m_ | R^2^_c_ |
| --- | --- | --- | --- | --- | --- | --- | --- |
| **PPH** | **~ UVB** | **4** | **-9.39** | **32.5** | **0** | **0.525** | **0.525** |
| MDH | ~ VPD | 4 | -10.71 | 35.1 | 2.66 | 0.530 | 0.705 |
| TMH | ~ Temp | 4 | -11.21 | 36.1 | 3.65 | 0.346 | 0.346 |
| Null | ~ intercept | 3 | -13.59 | 36.2 | 3.69 | 0 | 0 |
| TMH / MDH | ~ AWM size | 4 | -11.87 | 37.5 | 4.98 | 0.265 | 0.265 |
| MDH | ~ VPD + AWM size | 5 | -9.46 | 38.9 | 6.44 | 0.519 | 0.519 |
| TMH | ~ Temp + AWM size | 5 | -9.47 | 38.9 | 6.45 | 0.518 | 0.518 |

REFERENCES

Andersen, A. N., & Brault, A. (2010). Exploring a new biodiversity frontier: subterranean ants in northern Australia. *Biodiversity and Conservation*, 19, 2741–2750. doi:10.1007/s10531-010-9874-1

Beckmann, M., Václavík, T., Manceur, A. M., Šprtová, L., von Wehrden, H., Welk, E., & Cord, A. F. (2014). glUV: A global UV-B radiation data set for macroecological studies. *Methods in Ecology and Evolution*, 5, 372–383. doi:10.1111/2041-210X.12168

Bishop, T. R., Robertson, M. P., Gibb, H., van Rensburg, B. J., Braschler, B., Chown, S. L., … Parr, C. L. (2016). Ant assemblages have darker and larger members in cold environments. *Global Ecology and Biogeography*, 25, 1489–1499. doi:10.1111/geb.12516

Blaimer, B. B., Ward, P. S., Schultz, T. R., Fisher, B. L., & Brady, S. G. (2018). Paleotropical diversification dominates the evolution of the hyperdiverse ant tribe Crematogastrini (Hymenoptera: Formicidae). *Insect Systematics and Diversity*, 2, 1–14. doi:10.1093/isd/ixy013

Blomberg, S. P., Garland, T., & Ives, A. R. (2003). Testing for phylogenetic signal in comparative data: behavioral traits are more labile. *Evolution,* 57, 717–745. doi:10.1111/j.0014-3820.2003.tb00285.x

Moreau, C. S., & Bell, C. D. (2013). Testing the museum versus cradle tropical biological diversity hypothesis: phylogeny, diversification, and ancestral biogeographic range evolution of the ants. *Evolution*, 67, 2240–2257. doi:10.1111/evo.12105

Nelsen, M. P., Ree, R. H., & Moreau, C. S. (2018). Ant–plant interactions evolved through increasing interdependence. *Proceedings of the National Academy of Sciences*, 115, 12253–12258. doi:10.1073/pnas.1719794115

Pacheco, R., & Vasconcelos, H. L. (2012). Subterranean pitfall traps: Is it worth including them in your ant sampling protocol? *Psyche*, 870794. doi:10.1155/2012/870794

Pagel, M. (1999). Inferring the historical patterns of biological evolution. *Nature*, 401, 877–884. doi:10.1038/44766

Revell, L. J. (2012). phytools: an R package for phylogenetic comparative biology (and other things). *Methods in Ecology and Evolution*, *3*, 217–223. doi:10.1111/j.2041-210X.2011.00169.x

Ryder Wilkie, K. T., Mertl, A. L., & Traniello, J. F. A. (2007). Biodiversity below ground: Probing the subterranean ant fauna of Amazonia. *Naturwissenschaften*, 94, 725–731. doi:10.1007/s00114-007-0250-2

Schmidt, F. A., & Diehl, E. (2008). What is the effect of soil use on ant communities? *Neotropical Entomology*, 37, 381–388. doi:10.1590/S1519-566X2008000400005

Ward, P. S., Blaimer, B. B., & Fisher, B. L. (2016). A revised phylogenetic classification of the ant subfamily Formicinae (Hymenoptera: Formicidae), with resurrection of the genera Colobopsis and Dinomyrmex. *Zootaxa*, 4072, 343–357. doi:10.11646/zootaxa.4072.3.4

Yamaguchi, T., & Hasegawa, M. (1996). An experiment on ant predation in soil using a new bait trap method. *Ecological Research*, 11, 11–16. doi:10.1007/BF02347815

Yusah, K. M., Fayle, T. M., Harris, G., & Foster, W. A. (2012). Optimizing diversity assessment protocols for high canopy ants in tropical rain forest. *Biotropica*, 44, 73–81. doi:10.1111/j.1744-7429.2011.00775.x
